# Hidden diversity of soil giant viruses

**DOI:** 10.1038/s41467-018-07335-2

**Published:** 2018-11-19

**Authors:** Frederik Schulz, Lauren Alteio, Danielle Goudeau, Elizabeth M. Ryan, Feiqiao B. Yu, Rex R. Malmstrom, Jeffrey Blanchard, Tanja Woyke

**Affiliations:** 1 U.S. Department of Energy, Joint Genome Institute, Walnut Creek, CA USA; 2 Department of Biology, University of Massachusetts, Amherst, MA USA; 3 Department of Bioengineering, Stanford University, Stanford, CA USA

## Abstract

Known giant virus diversity is currently skewed towards viruses isolated from aquatic environments and cultivated in the laboratory. Here, we employ cultivation-independent metagenomics and mini-metagenomics on soils from the Harvard Forest, leading to the discovery of 16 novel giant viruses, chiefly recovered by mini-metagenomics. The candidate viruses greatly expand phylogenetic diversity of known giant viruses and either represented novel lineages or are affiliated with klosneuviruses, *Cafeteria roenbergensis* virus or tupanviruses. One assembled genome with a size of 2.4 Mb represents the largest currently known viral genome in the *Mimiviridae*, and others encode up to 80% orphan genes. In addition, we find more than 240 major capsid proteins encoded on unbinned metagenome fragments, further indicating that giant viruses are underexplored in soil ecosystems. The fact that most of these novel viruses evaded detection in bulk metagenomes suggests that mini-metagenomics could be a valuable approach to unearth viral giants.

## Abstract (web summary)

Current knowledge of giant virus diversity is largely based on samples from aquatic environments. Here the authors employ cultivation-independent metagenomics and mini-metagenomics on forest soil and identify 16 novel giant virus genomes, some of which representing novel lineages, including the so far largest genome in *Mimiviridae*.

## Introduction

Viruses larger than some cellular organisms and with genomes up to several megabases in size have been discovered in diverse environments across the globe, primarily from aquatic systems, such as freshwater, seawater and wastewater^1,2^, but also from terrestrial environments^3–5^ including permafrost^6,7^. These viruses are nucleocytoplasmic large DNA viruses (NCDLV), and they infect a wide range of eukaryotes, in particular protists and algae^8–11^. Only a few protist-infecting NCDLV have been recovered with their native hosts, such as *Cafeteria roenbergensis* virus (CroV) in the marine flagellate *Cafeteria roenbergensis*^12^ and the Bodo saltans virus (BsV)^13^. Many of the NCDLV are referred to as giant viruses based on their large physical size and a genome size of at least 300 kb^14^, although the term has also been applied to members of the NCLDV with genomes of at least 200 kb regardless of their particle size^15^. Importantly, for many of these NCDLV genome size and particle diameter do no correlate^8^.

Most of our current understanding of giant viruses comes from isolates retrieved in co-cultivation with laboratory strains of *Acanthamoeba*^1,3^. Only recently have the genomes of giant viruses been recovered by approaches, such as bulk shotgun metagenomics^16–20^, flow-cytometric sorting^21–23^, and after successful isolation using a wider range of protist hosts^23–25^. Recent large-scale marker gene-based environmental surveys^26–28^ hinted at an immense phylogenetic breadth of giant viruses of which, however, only a small fraction has been isolated to date. Possible reasons include challenges in providing a suitable host during co-cultivation and the inability to recover the viruses together with their native hosts^29^. In addition, a systematic recovery of giant virus genomes from metagenomic datasets is lacking and thus, the genetic diversity of giant viruses remains underexplored.

Here we describe 16 giant virus genomes from a forest soil ecosystem that were recovered using a cultivation-independent approach. We shed light on their coding potential and expand the phylogenetic framework of the NCLDV. Importantly, the novel genomes represent only the tip of the iceberg as revealed by a survey of the major capsid protein (MCP) encoded on unbinned metagenome fragments, which indicates a much higher untapped diversity of giant virus genetic material in soil.

## Results

### Mini-metagenomics facilitated the discovery of giant virus genomes

**Fig. 1:**
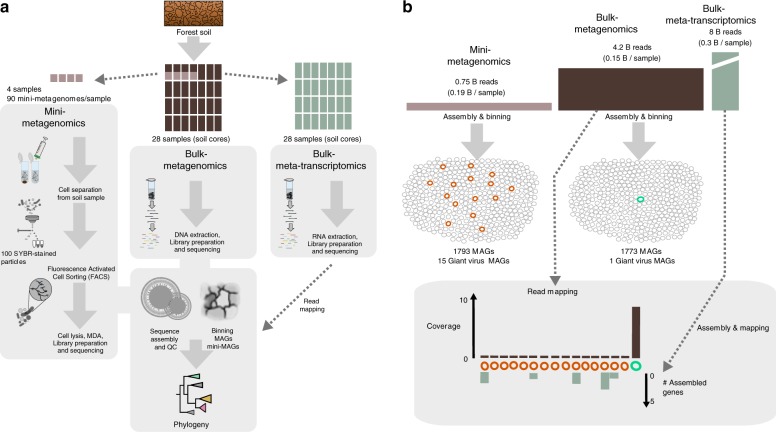
Discovery pipeline for soil giant viruses. **a** Overall workflow. Fourteen forest soil cores from Barre Woods long-term experimental warming site were sub-sampled into organic horizon and mineral zone resulting in 28 total samples. Total DNA and RNA were extracted from 28 soil samples for bulk metagenomics and metatranscriptomics. Of these samples, a subset of four encompassing two organic and two mineral layers were selected for flow-sorted mini-metagenomics. Cells and presumably viral particles, were separated from soil, stained with SYBR green nucleic acid stain and sorted using fluorescence activated cell sorting (FACS). Ninety sorted pools of 100 SYBR+ particles underwent lysis, whole genome amplification, library preparation, and sequencing on the Illumina NextSeq platform. Phylogenomic analysis of metagenome assembled genomes (MAGs) facilitated the identification of novel giant viruses. There was no correlation of presence or absence of giant viruses and sample treatment (Supplementary Table 3). **b** Data analysis summary. Fifteen giant virus MAGs (orange circles) were recovered from sorted samples, while only one giant virus MAG (turquoise circle) was recovered from the bulk metagenomes. The other 1778 MAGs from the mini-metagenomes (gray circles) and 1772 MAGs from the bulk metagenomes (gray circles) were of bacterial or archaeal origin and not analyzed further in this study. Mapping of bulk metagenome reads to MAGs revealed ~9× coverage of the bulk-metagenome derived MAG and <1× coverage of MAGs derived from mini-metagenomes, confirming the inability to recover these novel giant virus genomes using bulk metagenomics despite deep sequencing efforts. Assembly and mapping of metatranscriptome data indicated expression of only few of the novel giant virus genes of MAGs derived from mini-metagenomes

Soil samples from the Harvard Forest were subjected to standard shotgun sequencing of microbial communities. Four of the 28 samples were also analyzed using a ‘mini-metagenomics’^30–32^ approach, where multiple sets of 100 DNA-stained particles were flow sorted and subjected to whole genome amplification and sequencing (Fig. 1a). Metagenomic binning of assembled contigs produced 15 metagenome assembled genomes (MAGs) from the mini-metagenomes and 1 MAG from the bulk metagenomes (Supplementary Tables 1–4) that displayed features typically found in most NCLDV genomes^33,34^, such as hallmark genes encoding for MCP(s), factors for maturation of the viral capsid, and packaging ATPases (Supplementary Table 1, Supplementary Fig. 1). Furthermore, we observed on most contigs a uniform distribution of genes of viral, bacterial, or eukaryotic origin and many without matches in public databases (Supplementary Figs. 2, 3). In addition, these new viruses encoded numerous paralogous genes, a feature common to many NCLDV^35,36^ (Supplementary Fig. 2). Many of the duplicated genes were located on different contigs and often unique to the respective genomes, providing additional evidence that these contigs belong to a single viral MAG (Supplementary Fig. 1). Moreover, presence, absence, and copy number of nucleocytoplasmic virus orthologous genes (NCVOGs)^34^ were comparable to previously described giant viruses, suggesting that the MAGs are made up by single viral genomes and several of them being nearly complete (Supplementary Table 1, Supplementary Figs. 1, 4). An independently conducted benchmarking experiment of the mini-metagenomics approach revealed that no chimeric contigs are being created during this workflow which further supports the quality of the genomes derived here (Supplementary Figs. 5,  6).

Despite the bulk metagenome approach generating five-fold more reads, it only yielded in a single giant virus genome, whereas mini-metagenomics lead to the recovery of 15 additional bins attributable to NCLDV (Fig. 1b). Bulk metagenome reads only mapped to the MAG recovered from bulk metagenomes (at ~9× coverage) and not to any mini-metagenome MAGs, suggesting most of the discovered viruses were of low abundance in the sampled forest soil (Fig. 1b). This was also reflected in the soil metatranscriptomes in which no or only low transcriptional activity of the giant viruses could be detected (Fig. 1b, Supplementary Table 5).

### Sorted viral particles expand known diversity of NCLDV

**Fig. 2:**
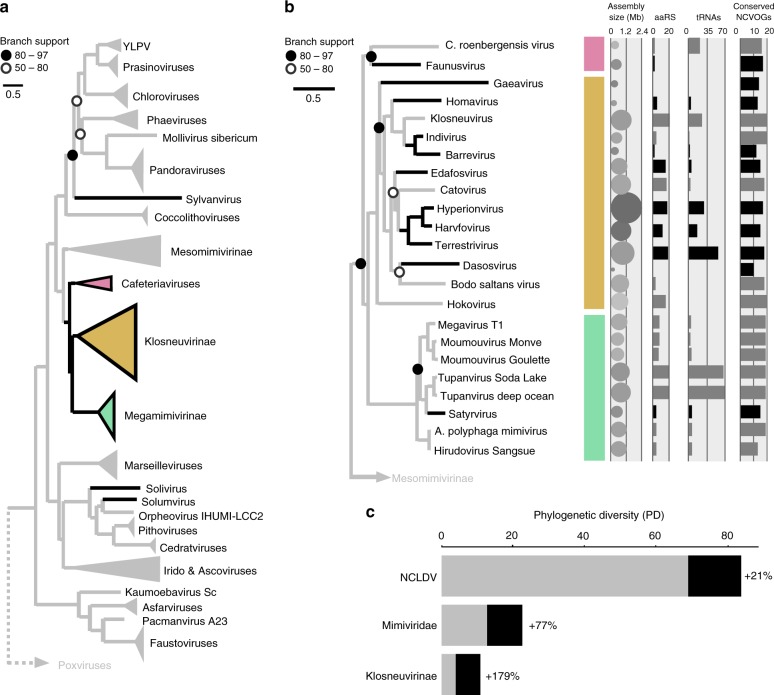
Expansion of NCLDV diversity by novel soil giant viruses. **a** Phylogenetic tree (IQ-tree LG+F+R6) of NCLDV inferred from a concatenated protein alignment of five core nucleocytoplasmic virus orthologous genes (NCVOGs)^34^. The tree was built from a representative set of NCDLV genomes after de-replication by ANI clustering (95% id). Novel soil NCLDV lineages and existing major NCLDV lineages grouping together with soil NCLDV are highlighted in black. The scale bar represents substitutions per site. Branch support values are shown in data S1. Branches are collapsed if support was low (<50), filled circles indicate moderate support (50–80, white) or high support (80–97, black), branches without circles are fully supported (>97). **b** Detailed phylogenetic tree of the *Mimiviridae*. Diameter of filled circles correlates with assembly size and shades of gray with GC% ranging from 20% (light gray) to 60% (dark gray). Bar plots summarize total number of encoded aminoacyl-tRNA synthetases (aaRS) and tRNAs. In addition, completeness was estimated based on number of identified marker genes out of 20 ancestral NCVOGs (more details are shown in Supplementary Fig. 1). **c** Increase of phylogenetic diversity (PD) after adding the soil NCLDV MAGs (black) to representative sets of NCLDV reference genomes (gray). Naming considerations are shown in Supplementary Table 2

The phylogenetic relationships inferred from the tree built from a concatenated alignment of five core NCVOGs^34,37^ (Fig. 2a; Supplementary Fig. 1) and the consensus of single protein phylogenies (Supplementary Figs. 7, 8) showed that newly discovered viruses from forest soil were affiliated with diverse lineages in the NCLDV. Two of the new viruses, solivirus, and solumvirus, were in sister-position to the pithoviruses, cedratviruses and the recently isolated orpheovirus^38^. Sylvanvirus represented a long branch on its own. Most novel soil NCLDV were positioned within the family *Mimiviridae*, which comprises the proposed subfamilies *Megamimivirinae*, the *Klosneuvirinae*, the algae-infecting *Mesomimivirinae* and the genus *Cafeteriavirus*^17,39^ (Fig. 2b). One of the new viruses, faunusvirus, grouped with CroV and represents the second viral genome sampled in this clade (Fig. 2b). Another novel virus, satyrvirus, branched as sister lineage to the two recently isolated tupanviruses, which were derived from deep sea and a soda lake samples^9^, together forming a monophyletic clade in the *Megamimivirinae* (Fig. 2b). Thus, satyrvirus can be considered as a third member of the proposed genus *Tupanvirus*^40^. Notably, none of the new lineages were directly affiliated with any of the three other subgroups of well-studied *Megamimivirinae*^41,42^. Eight of the new viruses branched within the proposed *Klosneuvirinae*, currently the largest subfamily in the *Mimiviridae* based on phylogenetic diversity (PD)^43^ (Fig. 2c).

Strikingly, the addition of the novel giant viruses to the NCLDV tree lead to a 21% increase of the total PD in the NCLDV (Fig. 2c), expanded the diversity of the *Mimiviridae* by 77% and nearly tripled the PD of the *Klosneuvirinae* (Fig. 2c). It is important to note that this expansion of PD was from a single study using cultivation-independent techniques, thereby building upon decades of previous giant virus discovery work^1,8,10,41^. The fact that all these newly discovered viruses represent distinct lineages in the NCLDV hints that additional sampling is expected to lead to a further substantial increase in giant virus PD.

### Genomic features of soil giant viruses

The assembled viral genomes assigned to the klosneuviruses were among the largest ever found (Fig. 2b; Supplementary Fig. 1, Supplementary Table 1). With a genome size of up to 2.4 Mb the hyperionvirus would become the new record for genome size in the *Mimiviridae*, dwarfing klosneuvirus and tupanvirus with their ~ 1.5 Mb genomes^9,17^. Considering that several of the forest soil MAGs are potentially only partially complete, the true genome size of the new viruses might be even larger. Similar to recently discovered klosneuviruses and tupanviruses^9,17^, several of the new viruses affiliated with the *Klosneuvirinae* encode for expanded sets of aminoacyl tRNA synthetases (aaRS), e.g. terrestrivirus with up to 19 different aaRS and up to 50 tRNAs with specificity for all 20 different amino acids, a feature only very recently described in the tupanviruses^9^. In concert with other viral components of the eukaryotic translation system, such viruses likely override host protein biosynthesis using their own enzymes to ensure efficient production of viral proteins. Being less dependent on the host cell machinery might allow these viruses to infect multiple hosts, i.e. fewer proteins are necessary to target and interact with alternative hosts. A broader host range has been experimentally verified for tupanviruses^9^ which were able to infect different protists, however, viral titer did not increase in all the cases^9^.

### Genome novelty of soil giant viruses

**Fig. 3:**
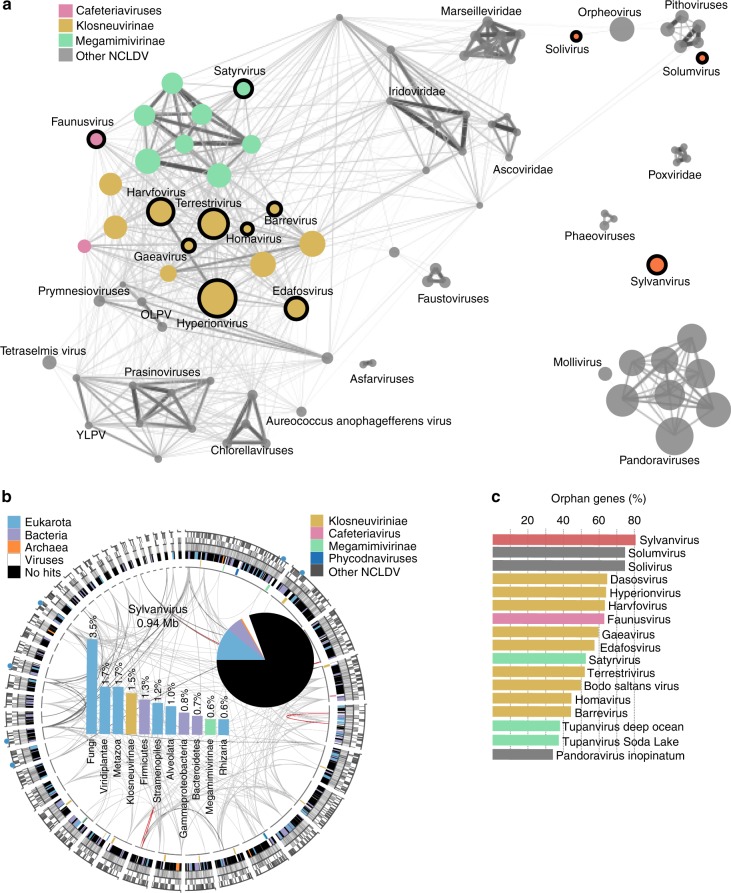
Genome novelty of soil giant viruses. **a** Nucleocytoplasmic large DNA virus (NCLDV) gene sharing network, with nodes representing genomes, node diameter correlating with genome size, edge diameter and color intensity with normalized percentage of genes in shared gene families between node pairs above a threshold of 18%. **b** Circular representation of the sylvanvirus genome. From outside to inside: Blue filled circles depict location of encoded tRNAs. The second ring displays positions of genes (gray) either on the minus or the plus strand. The next track illustrates GC content in shades of gray ranging from 20% (white) to 60% (dark gray). The fourth track shows color-coded origin of proteins with best blastp hits (*e*-value 1e−5) to cellular homologs. Best hits against viral proteins are indicated in white and if possible, further broken down based on their taxonomic origin color-coded on the most inner track. Finally, lines in the middle of the plot connect paralogs (gray) and nearly identical repeats (orange). The pi chart in the center of the plot summarizes the percentage of genes with and without cellular homologs, which are further broken down based on best blastp hits (*e*-value 1e−5) hits in the adjacent bar plot. **c** Percentage of genes in NCLDV genomes with bacterial or eukaryotic homologs and with no blastp hits (*e*-value 1e−5) in the NCBI nr database, highlighting the unique position of sylvanvirus

Complementary to the phylogenetic analysis (Fig. 2a), we inferred a gene sharing network to provide further insights into the relationship of the novel viral genomes to known NCLDV lineages based on shared gene content. In agreement with the species tree, viral lineages such as the *Mimiviridae*, the *Marseilleviridae*, the pithoviruses and cedratviruses, the faustoviruses and the molliviruses and pandoraviruses remained well connected (Fig. 3a). Among the novel viruses with the lowest percentage of genes shared with other NCLDV were solumvirus and solivirus, with solivirus being only connected to orpheovirus and *Marseilleviridae* and solumvirus to the cedratviruses. In contrast to the phylogenetic tree in which solivirus and solumvirus were affiliated to each other, there was no particular linkage between them in the network. This suggests limited taxon sampling and we expect that with discovery of additional giant virus genomes, the phylogenetic position of these viruses will be better resolved.

Another of the soil giant viruses denoted as sylvanvirus featured a genome completely disconnected from all other NCLDV (Fig. 3a). With a size of almost 1 Mb it represents one of the largest viral genomes outside pandoraviruses and the *Mimiviridae* (Fig. 3a; Supplementary Fig. 1)^8,44^. With the presence of 10 ancestral NCLDV genes, a number similar to several other NCLDV, the sylvanvirus genome can be considered near complete (Supplementary Fig. 1). Intriguingly, the vast majority (~80%) of its proteins had neither matches in the NCBI non-redundant (nr) database (Fig. 3b). From the proteins with database hits, 57% had matches to eukaryotes and 27% to bacteria but only 13% to other viruses (Fig. 3c). Importantly, there was no trend in taxonomic affiliation of the hits (Fig. 3c), again emphasizing the lack of any affiliation to known viruses and organisms. Among the identifiable genes were 18 potential kinases, five ubiquitin ligases, and a histone, all potentially playing important roles in interaction with a currently unknown host.

### True diversity of giant viruses in forest soil

**Fig. 4:**
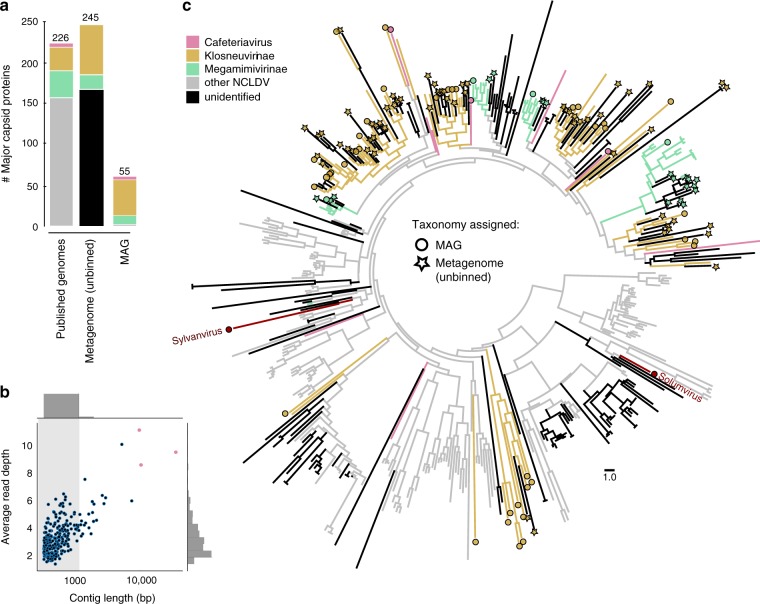
Hidden diversity of giant viruses in bulk metagenomes. **a** Total number of major capsid proteins (MCPs) found in reference nucleocytoplasmic large DNA virus (NCLDV) genomes, metagenome assembled genomes (MAGs), or recovered from bulk metagenomes on contigs >1 kb and contigs <1 kb (dark gray), colored by taxonomy. **b** Size and cover of bulk metagenome contigs containing MCP genes, either from the unbinned fraction (filled blue circles) or the MAGs (filled pink circles). **c** Phylogenetic tree of the MCPs of NCLDV. Branches are color-coded based on taxonomic origin of MCPs inferred by relationship in the tree to MCPs of known reference NCLDV. MCPs of novel giant viruses from this study which are not members of the *Mimiviridae* are indicated in red. Branches labeled with a circle represent novel MCP from MAGs generated in this study while stars indicate MCPs recovered from the unassembled fraction (contigs >1 kb) of bulk-metagenomes. Circles and stars are filled in color if taxonomy could be assigned based on the tree and in black if it was not possible to assign taxonomy

The MCPs in the bulk metagenomes revealed that the 16 novel viral genomes represent just a small fraction of giant virus diversity in the soil samples (Fig. 4a). In total, 245 different MCP genes were detected, of which 99% were part of the unbinned metagenome fraction. Most of these MCPs were located on short contigs with a read coverage of below 2, indicating an extremely low abundance of corresponding NCLDV in the respective samples (Fig. 4b). Importantly, none of the bulk-metagenome MCPs matched MCPs from the mini-metagenome-derived MAGs, further underlining the much greater diversity of giant viruses in these samples. MCPs can be heavily duplicated but usually branch together in lineage-specific clades enabling taxonomic classification based on their nearest neighbors in the tree^45^. Based on identified phylogenetic relationships it was possible to assign taxonomy to several of the bulk metagenome MCPs, of which most could be attributed to the klosneuviruses (Fig. 4a, c). A hint of the true dimension of the NCLDV diversity is revealed when considering that the total number of nearly 300 MCPs discovered in this study, which includes MCPs from all the MAGs, exceeds the 226 MCPs identified in previously published NCLDV genomes.

## Discussion

Our results illustrate that employing cultivation independent methods on a minute sample from forest soil, a habitat in which giant viruses have rarely been found previously^3,45^, can lead to key discoveries. Recovery of solumvirus, solivirus, and sylvanvirus, three potentially genus, subfamily, or even family level NCLDV lineages together with 13 other novel giant virus genomes vastly expands the PD of the NCLDV and provides new insights into their genetic makeup.

The fact that only a single giant virus MAG was recovered in the bulk metagenomes suggests extremely low abundance of these viruses compared to bacterial and archaeal community members in forest soil. However, mini-metagenomics has proven most effective in recovering these viruses, yet without any detectable traces of host sequences (Supplementary Tables 6, 7). It is noteworthy that oftentimes the average read coverage of the giant virus MAGs was the highest or among the highest compared to non-viral MAGs derived from the same mini-metagenomes pool of 100 DNA-stained particles (Supplementary Fig. 9). The high coverage and completeness of giant virus genomes is consistent with having several copies of the same viral genome in the same mini-metagenome pool, but the overall low abundance of giant viruses in the system makes it unlikely that several identical viral particles were sorted by chance (Supplementary Figs. 1, 9). A plausible scenario could be that host vacuoles already filled with giant viruses may have been recovered during sorting, thereby delivering several clonal copies of a giant virus genome into a single mini-metagenome pool. This would enable genome assembly of higher quality and completeness, as previously shown for polyploid bacterial symbionts^46^.

Of the few available studies that have used this mini-metagenomes method, one describes the discovery of a novel intracellular bacterium^30^ and another a new group of giant viruses^17^, suggesting mini-metagenomics is a compelling method for elucidating the hidden diversity of intracellular entities such as giant viruses. As shown by the MCP diversity in the unbinned metagenome fraction many novel giant viruses are readily awaiting discovery. Importantly, the mini-metagenomics approach has not been exhaustively performed in soil or any other ecosystem and thus represents a promising addition to the toolkit for exploring the untapped diversity in the giant virus universe.

## Methods

### Sampling and sample preparation

Fourteen forest soil cores from the Barre Woods warming experiment located at the Harvard Forest Long-Term Ecological Research site (Petersham, MA) were collected and sub-sampled into organic horizon and mineral zone, resulting in 28 total samples. Mineral zone samples were flash-frozen while organic horizons were incubated with deuterium oxide for 2 weeks prior to freezing to label the active bacterial and archaeal communities. This incubation was carried out as part of a different experiment that will be addressed in a later manuscript. Total DNA and RNA were extracted from 28 soil samples for bulk metagenomics and metatranscriptomics using the MoBio PowerSoil DNA and RNA kits, respectively. Bacterial and Plant rRNA depletion was performed on the RNA samples prior to sequencing. Of these 28 soil samples, a subset of four encompassing two organic and two mineral layers were selected for mini-metagenomics. Cells, and presumably viral particles and/or eukaryote vacuoles containing them, were separated from soil particles using a mild detergent, followed by vortexing, centrifugation, and filtration through a 5 μm syringe filter. The filtrates were stained with SYBR Green nucleic acid stain. For each of the four samples, 90 pools containing 100 SYBR+ particles were sorted into microwell plates using fluorescence activated cell sorting (FACS). Sorted pools underwent lysis and whole genome amplification through multiple displacement amplification (MDA) following methods outlined previously^47^. A total of 360 sequencing libraries were generated with the Nextera XT v2 kit (Illumina) with 9 rounds of PCR amplification.

### Mini-metagenomes

The 360 libraries derived from sorted particles were sequenced at the DOE Joint Genome Institute (JGI, Walnut Creek, CA) using the Illumina NextSeq platform. Pools of 90 libraries were processed in four sequencing runs that generated 2 × 150 bp read lengths. Raw Illumina reads were quality filtered to remove contamination and low-quality reads using BBTools (http://bbtools.jgi.doe.gov, version 37.38). Read normalization was performed using BBNorm (http://bbtools.jgi.doe.gov) and error correction with Tadpole (http://bbtools.jgi.doe.gov). Assembly of filtered, normalized Illumina reads was performed using SPAdes (v3.10.1)^48^ with the following options:–phred-offset 33 -t 16 -m 115–sc -k 25,55,95. All contig ends were then trimmed of 200 bp and contigs were discarded if the length was <2 kb or read coverage <2 using BBMap (http://bbtools.jgi.doe.gov) with the following options: nodisk ambig, filterbycoverage.sh: mincov.

### Bulk metagenomes

Unamplified TruSeq libraries were prepared for the 28 DNA samples for metagenomic sequencing on the Illumina HiSeq-2000 platform at the DOE JGI. Raw Illumina reads were trimmed, quality filtered, and corrected using bfc (version r181)^49^ with the following options: -1 -s 10g -k 21 -t 10. Following quality filtering, reads were assembled using SPAdes (v3.11.1)^48,50^ with the following options:-m 2000–only-assembler -k 33,55,77,99,127–meta -t 32. The entire filtered read set was mapped to the final assembly and coverage information generated using bbmap (http://bbtools.jgi.doe.gov, version 37.62) with default parameters except ambiguous = random. The version of the processing pipeline was jgi_mga_meta_rqc.py, 2.1.0.

### Metatranscriptomes

Libraries were prepared and sequenced on the Illumina NextSeq platform at the DOE JGI. Following sequencing, metatranscriptome reads were quality cleaned and a combined assembly was generated using the MEGAHIT assembler (v1.1.2)^51^ using the following options: -m 0.2—k-list 23,43,63,83,103,123—continue -o out.megahit—12. These cleaned reads were aligned to metagenome reference sequences using BBMap (http://bbtools.jgi.doe.gov, version 37.38) with the following options: nodisk = true interleaved = true ambiguous = random.

### Metagenome binning

Contigs were organized into genome bins based on tetranucleotide sequence composition with MetaBat2^52^. Genome bins were generated for mini-metagenomes without contig coverage patterns due to MDA bias^53^. Coverage was determined for the bulk metagenomes by mapping reads to the completed assemblies using the Burrows–Wheeler aligner^54^. Taxonomy of bins was determined with the genome taxonomy database classifier (https://github.com/Ecogenomics/GTDBTk).

### Screening for giant viruses

Metagenomic bins were screened for presence of the 20 ancestral NCVOGs^34^ with hmmsearch (version 3.1b2, hmmer.org). Bins with more than five different hits and/or that contained the NCLDV MCP gene (NCVOG0022) were selected and further evaluated (see below).

### Annotation and quality control of viral genome bins

Gene calling was performed with GeneMarkS using the virus model^55^. For functional annotation proteins were blasted against previously established NCVOGs^34^ and the NCBI non-redundant database (nr) using Diamond blastp^56^ with an *e*-value cutoff of 1.0e−5. In addition, protein domains were identified by hmmsearch (version 3.1b2, hmmer.org) against Pfam-A (version 29.0)^57^, and tRNAs and introns were identified using tRNAscan-SE^58^ and cmsearch from the Infernal package^59^ against the Rfam database (version 13.0)^60^. Nearly identical sequences within genome bins (>100 bp, identity >94%) were detected using the MUMmer repeat-match algorithm^61^ and visualized with Circos^62^ together with the respective genome bins. For all MAGs, paralogs and best diamond blastp vs. NCBI nr hits were visualized with Circos^62^. Furthermore, distribution of read depth across contigs was evaluated and regions with low average coverage were identified (Supplementary Table 4).

### Experimental benchmarking of the mini-metagenomics approach

Benchmarking of the mini-metagenomics approach to assess potential chimera formation during MDA was performed by randomly sorting 10 cells from a bacterial mock community consisting of five different bacterial isolates; *Escherichia coli* K12, *Echinicola vietnamensis* DSM 17526, *Shewanella oneidensis* MR-1, *Pseudomonas putida* F1, and *Meiothermus ruber*. In total 59 of these 10-cell sorts were subject to MDA and sequencing. Resulting reads were filtered, assembled and analyzed with the same bioinformatics pipeline used for the mini-metagenomes generated in this study. Assembly statistics of recovered MAGs were generated with MetaQUAST^63^.

### Computational benchmarking of giant virus metagenomic binning

In addition, benchmarking of the binning workflow was performed to assess its applicability to giant virus data. First, binning of a simulated mock community consisting of 12 giant viruses was tested, each a representative of a subfamily or family in the NCLDV. In addition, the herein newly discovered giant viruses were used as template for a second simulated mock community. In brief, MDA was simulated on the genomes of the mock communities with MDAsim^64^ (https://github.com/hzi-bifo/mdasim/releases/v2.1.1). In the following, Illumina reads were generated with ART^65^ and the same bioinformatics pipeline used for the mini-metagenomes in this study employed for read error-correction, normalization, assembly, and binning.

### Phylogenomics

To remove redundancy, the set of 186 published NCLDV genomes and 16 novel soil giant viruses were clustered at an average nucleotide identity (ANI) of 95% with at least 100 kb-aligned fraction using fastANI^66^ resulting in 132 clusters and singletons. None of the newly discovered viruses clustered with any other virus. The three most incomplete novel giant virus genomes were removed from the data set (Supplementary Table 1, Supplementary Fig. 2). To infer the positions of novel soil giant viruses in the NCLDV, five core NCLDV proteins^34^ were selected: DNA polymerase elongation subunit family B (NCVOG0038), D5-like helicase-primase (NCVOG0023), packaging ATPase (NCVOG0249), and DNA or RNA helicases of superfamily II (NCVOG0076) and Poxvirus Late Transcription Factor VLTF3-like (NCVOG0262), and identified with hmmsearch (version 3.1b2, hmmer.org). Three of the MAGs derived from mini-metagenomes were excluded from the analysis as they had less than three conserved NCLDV proteins (Supplementary Table 1). Protein sequences were aligned using mafft^67^. Gapped columns in alignments (<10% sequence information) and columns with low information content were removed from the alignment with trimal^68^. Phylogenetic trees for each protein and for a concatenated alignment of all five proteins were constructed using IQ-tree with LG+F+R6 as suggested by model test as best-fit substitution model^69^. The percentage increase in PD^41^ was calculated based on the difference of the sum of branch lengths of phylogenetic species of the NCLDV trees with and without the metagenomic soil giant viruses.

### MCP analysis

Bulk metagenome assemblies and 186 published NCLDV genomes and 16 soil MAGs were screened for presence of the NCLDV MCP gene (NCVOG0022)^17,34^ with hmmsearch (version 3.1b2, hmmer.org) and applying a cutoff of 1e−6. This cutoff has been evaluated against ~60,000 available bacterial, archaeal, eukaryotic, and other non-NCLDV genomes in the Integrated Microbial Genomes database^70^ yielding in only few false positives. Resulting protein hits were extracted from the metagenome and to reduce redundancy clustered with cd-hit at a sequence similarity of 95%^71^. Cluster representatives were then subject to diamond blastp^56^ against nr database (June 2018) and proteins which had hits but no NCLDV MCP in the top 10 were excluded from further analysis as potentially false positives. For tree construction, MCPs were extracted and aligned with mafft-ginsi (–unalignlevel 0.8,–allowshift)^67^. Gapped columns in the alignment (<10% sequence information) were removed with trimal^68^ and proteins with <50 aligned amino acids were removed. A phylogenetic tree was constructed with IQ-tree and the LG+F+R8 as suggested by model test as the best-fit substitution model^69^.

### Gene sharing network

Protein families were inferred with OrthoFinder 1.03^72^ on a representative dataset of 93 NCLDV genomes for comparative analysis (after de-replication using 95% ANI clustering^66^, details described above, and removal of 36 poxviruses). For each pair of NCLDV genomes (ANI 95% cluster representatives) the average percentage of proteins in shared orthogroups in relation to the total number of proteins in the respective genome was calculated and used as edge weight in the network.The network was created in Gephi^73^ using a force layout and filtered at an edge weight of 18%.

## Electronic supplementary material

Supplementary Information

## Data Availability

The giant virus genomes were deposited at NCBI Genbank (MK071979–MK072551) and at https://bitbucket.org/berkeleylab/forestsoil-gv, together with sequence alignments and phylogenetic trees underlying this study. Metagenomes and corresponding metadata are available at https://img.jgi.doe.gov/m, accession numbers indicated in Supplementary Table 3.

## References

[1] Aherfi S, Colson P, La Scola B, Raoult D (2016). Giant viruses of amoebas: an update. Front. Microbiol.

[2] Andrade ACDSP (2018). Ubiquitous giants: a plethora of giant viruses found in Brazil and Antarctica. Virol. J..

[3] Pagnier I (2013). A decade of improvements in Mimiviridae and Marseilleviridae isolation from amoeba. Intervirology.

[4] Yoosuf N (2014). Draft genome sequences of Terra1 and Terra2 viruses, new members of the family Mimiviridae isolated from soil. Virology.

[5] Boughalmi M (2013). High-throughput isolation of giant viruses of the Mimiviridae and Marseilleviridae families in the Tunisian environment. Environ. Microbiol..

[6] Legendre M (2015). In-depth study of Mollivirus sibericum, a new 30,000-y-old giant virus infecting Acanthamoeba. Proc. Natl Acad. Sci. USA.

[7] Legendre M (2014). Thirty-thousand-year-old distant relative of giant icosahedral DNA viruses with a pandoravirus morphology. Proc. Natl Acad. Sci. USA.

[8] Abergel C, Legendre M, Claverie JM (2015). The rapidly expanding universe of giant viruses: Mimivirus, Pandoravirus, Pithovirus and Mollivirus. FEMS Microbiol. Rev..

[9] Abrahão J (2018). Tailed giant Tupanvirus possesses the most complete translational apparatus of the known virosphere. Nat. Commun..

[10] Fischer MG (2016). Giant viruses come of age. Curr. Opin. Microbiol..

[11] Wilson WH, Van Etten JL, Allen MJ (2009). The Phycodnaviridae: the story of how tiny giants rule the world. Curr. Top. Microbiol. Immunol..

[12] Fischer MG, Allen MJ, Wilson WH, Suttle CA (2010). Giant virus with a remarkable complement of genes infects marine zooplankton. Proc. Natl Acad. Sci. USA.

[13] Deeg, C. M., Chow, C.-E. T. & Suttle, C. A. The kinetoplastid-infecting Bodo saltans virus (BsV), a window into the most abundant giant viruses in the sea. *eLife* **7**, e33014 (2018). 10.7554/eLife.33014 PMC5871332 29582753

[14] Claverie JM, Abergel C (2016). Giant viruses: the difficult breaking of multiple epistemological barriers. Stud. Hist. Philos. Biol. Biomed. Sci..

[15] Wilhelm Steven, Bird Jordan, Bonifer Kyle, Calfee Benjamin, Chen Tian, Coy Samantha, Gainer P., Gann Eric, Heatherly Huston, Lee Jasper, Liang Xiaolong, Liu Jiang, Armes April, Moniruzzaman Mohammad, Rice J., Stough Joshua, Tams Robert, Williams Evan, LeCleir Gary (2017). A Student’s Guide to Giant Viruses Infecting Small Eukaryotes: From Acanthamoeba to Zooxanthellae. Viruses.

[16] Verneau J, Levasseur A, Raoult D, La Scola B, Colson P (2016). MG-Digger: an automated pipeline to search for giant virus-related sequences in metagenomes. Front. Microbiol..

[17] Schulz F (2017). Giant viruses with an expanded complement of translation system components. Science.

[18] Zhang W (2015). Four novel algal virus genomes discovered from Yellowstone Lake metagenomes. Sci. Rep..

[19] Andreani J, Verneau J, Raoult D, Levasseur A, La Scola B (2018). Deciphering viral presences: two novel partial giant viruses detected in marine metagenome and in a mine drainage metagenome. Virol. J..

[20] Roux S (2017). Ecogenomics of virophages and their giant virus hosts assessed through time series metagenomics. Nat. Commun..

[21] Wilson WH (2017). Genomic exploration of individual giant ocean viruses. ISME J..

[22] Martínez Martínez J, Swan BK, Wilson WH (2014). Marine viruses, a genetic reservoir revealed by targeted viromics. ISME J..

[23] Khalil JYB (2016). High-throughput isolation of giant viruses in liquid medium using automated flow cytometry and fluorescence staining. Front. Microbiol..

[24] Bajrai LH (2016). Kaumoebavirus, a new virus that clusters with faustoviruses and Asfarviridae. Viruses.

[25] Reteno DG (2015). Faustovirus, an asfarvirus-related new lineage of giant viruses infecting amoebae. J. Virol..

[26] Mihara Tomoko, Koyano Hitoshi, Hingamp Pascal, Grimsley Nigel, Goto Susumu, Ogata Hiroyuki (2018). Taxon Richness of “Megaviridae” Exceeds those of Bacteria and Archaea in the Ocean. Microbes and Environments.

[27] Colson P, Aherfi S, La Scola B (2017). Evidence of giant viruses of amoebae in the human gut. Hum. Microbiome J..

[28] Hingamp P (2013). Exploring nucleo-cytoplasmic large DNA viruses in Tara Oceans microbial metagenomes. ISME J..

[29] Halary S, Temmam S, Raoult D, Desnues C (2016). Viral metagenomics: are we missing the giants?. Curr. Opin. Microbiol..

[30] McLean JS (2013). Candidate phylum TM6 genome recovered from a hospital sink biofilm provides genomic insights into this uncultivated phylum. Proc. Natl Acad. Sci. USA.

[31] Yu, F. B. et al. Microfluidic-based mini-metagenomics enables discovery of novel microbial lineages from complex environmental samples. *eLife* **6**, e26580 (2017). 10.7554/eLife.26580 PMC5498146 28678007

[32] Berghuis, B. A. et al. Hydrogenotrophic methanogenesis in archaeal phylum Verstraetearchaeota reveals the shared ancestry of all methanogens. Preprint at 10.1101/391417 (2018). 10.1073/pnas.1815631116 PMC6421429 30814220

[33] Iyer LM, Aravind L, Koonin EV (2001). Common origin of four diverse families of large eukaryotic DNA viruses. J. Virol..

[34] Yutin N, Wolf YI, Raoult D, Koonin EV (2009). Eukaryotic large nucleo-cytoplasmic DNA viruses: clusters of orthologous genes and reconstruction of viral genome evolution. Virol. J..

[35] Filée J (2013). Route of NCLDV evolution: the genomic accordion. Curr. Opin. Virol..

[36] Suhre K (2005). Gene and genome duplication in Acanthamoeba polyphaga Mimivirus. J. Virol..

[37] Yutin N, Wolf YI, Koonin EV (2014). Origin of giant viruses from smaller DNA viruses not from a fourth domain of cellular life. Virology.

[38] Andreani J (2017). Orpheovirus IHUMI-LCC2: a new virus among the iant viruses. Front. Microbiol.

[39] Gallot-Lavallée, L., Blanc, G. & Claverie, J.-M. Comparative genomics of *Chrysochromulina ericina*virus and other microalga-infecting large DNA viruses highlights their intricate evolutionary relationship with the established Mimiviridae family. *J. Virol*. **91**, e00230-17 (2017). 10.1128/JVI.00230-17 PMC5487555 28446675

[40] Rodrigues, R. A. L., Mougari, S., Colson, P., La Scola, B. & Abrahão, J. S. ‘Tupanvirus’, a new genus in the family Mimiviridae. *Arch. Virol*. doi: 10.1007/s00705-018-4067-4 (2018). 10.1007/s00705-018-4067-4 30291500

[41] Colson P, La Scola B, Levasseur A, Caetano-Anollés G, Raoult D (2017). Mimivirus: leading the way in the discovery of giant viruses of amoebae. Nat. Rev. Microbiol..

[42] La Scola B (2003). A giant virus in amoebae. Science.

[43] Wu D (2009). A phylogeny-driven genomic encyclopaedia of Bacteria and Archaea. Nature.

[44] Legendre M (2018). Diversity and evolution of the emerging Pandoraviridae family. Nat. Commun..

[45] Wilhelm SW, Coy SR, Gann ER, Moniruzzaman M, Stough JMA (2016). Standing on the shoulders of giant viruses: five lessons learned about large viruses infecting small eukaryotes and the opportunities they create. PLoS Pathog..

[46] Woyke T (2010). One bacterial cell, one complete genome. PLoS One.

[47] Rinke C (2014). Obtaining genomes from uncultivated environmental microorganisms using FACS-based single-cell genomics. Nat. Protoc..

[48] Bankevich A (2012). SPAdes: a new genome assembly algorithm and its applications to single-cell sequencing. J. Comput. Biol..

[49] Li H (2015). BFC: correcting Illumina sequencing errors. Bioinformatics.

[50] Nurk S, Meleshko D, Korobeynikov A, Pevzner P (2017). A. metaSPAdes: a new versatile metagenomic assembler. Genome Res..

[51] Li D (2016). MEGAHIT v1.0: a fast and scalable metagenome assembler driven by advanced methodologies and community practices. Methods.

[52] Kang DD, Froula J, Egan R, Wang Z (2015). MetaBAT, an efficient tool for accurately reconstructing single genomes from complex microbial communities. PeerJ.

[53] Woyke T (2009). Assembling the marine metagenome, one cell at a time. PLoS One.

[54] Li H, Durbin R (2009). Fast and accurate short read alignment with Burrows-Wheeler transform. Bioinformatics.

[55] Borodovsky, M. & Lomsadze, A. Gene identification in prokaryotic genomes, phages, metagenomes, and EST sequences with GeneMarkS suite. *Curr. Protoc. Bioinformatics* **27**, 3911 (2002). 10.1002/0471250953.bi0405s35 21901741

[56] Buchfink B, Xie C, Huson DH (2015). Fast and sensitive protein alignment using DIAMOND. Nat. Methods.

[57] Finn RD (2016). The Pfam protein families database: towards a more sustainable future. Nucleic Acids Res..

[58] Lowe TM, Chan PP (2016). tRNAscan-SE On-line: integrating search and context for analysis of transfer RNA genes. Nucleic Acids Res..

[59] Nawrocki EP, Eddy SR (2013). Infernal 1.1: 100-fold faster RNA homology searches. Bioinformatics.

[60] Kalvari I (2018). Rfam 13.0: shifting to a genome-centric resource for non-coding RNA families. Nucleic Acids Res..

[61] Delcher AL, Salzberg SL, Phillippy AM (2003). Using MUMmer to identify similar regions in large sequence sets. Curr. Protoc. Bioinforma..

[62] Krzywinski M (2009). Circos: an information aesthetic for comparative genomics. Genome Res..

[63] Mikheenko A, Saveliev V, Gurevich A (2016). MetaQUAST: evaluation of metagenome assemblies. Bioinformatics.

[64] Tagliavi, Z. & Draghici, S. MDAsim: a multiple displacement amplification simulator. In *2012 IEEE International Conference on Bioinformatics and Biomedicine (BIBM)* 1–4 (IEEE, 2012).

[65] Huang W, Li L, Myers JR, Marth GT (2012). ART: a next-generation sequencing read simulator. Bioinformatics.

[66] Jain, C., Rodriguez-R. L. M., Phillippy, A. M., Konstantinidis, K. T. & Aluru, S. High-throughput ANI analysis of 90K prokaryotic genomes reveals clear species boundaries. Preprint at https://www.biorxiv.org/content/early/2017/11/27/225342 (2017). 10.1038/s41467-018-07641-9 PMC6269478 30504855

[67] Katoh K, Standley DM (2016). A simple method to control over-alignment in the MAFFT multiple sequence alignment program. Bioinformatics.

[68] Capella-Gutiérrez S, Silla-Martínez JM, Gabaldón T (2009). trimAl: a tool for automated alignment trimming in large-scale phylogenetic analyses. Bioinformatics.

[69] Nguyen LT, Schmidt HA, von Haeseler A, Minh BQ (2015). IQ-TREE: a fast and effective stochastic algorithm for estimating maximum-likelihood phylogenies. Mol. Biol. Evol..

[70] Chen IMA (2017). IMG/M: integrated genome and metagenome comparative data analysis system. Nucleic Acids Res..

[71] Li W, Godzik A (2006). Cd-hit: a fast program for clustering and comparing large sets of protein or nucleotide sequences. Bioinformatics.

[72] Emms DM, Kelly S (2015). OrthoFinder: solving fundamental biases in whole genome comparisons dramatically improves orthogroup inference accuracy. Genome Biol..

[73] Bastian M, Heymann S, Jacomy M (2009). Gephi: an open source software for exploring and manipulating networks. Icwsm.

