# Supplementary material for: Hidden diversity of soil giant viruses
Source: Nat Commun. 2018 Nov 19;9:4881. doi: 10.1038/s41467-018-07335-2 (PMC6243002; doi:10.1038/s41467-018-07335-2)
Supplement: Supplementary file 1 — Supplementary Information [file 41467_2018_7335_MOESM1_ESM.pdf]

Hidden diversity of soil giant viruses

Frederik Schulz, Lauren Alteio, Danielle Goudeau, Elizabeth M. Ryan, Feiqiao B. Yu, Rex R. Malmstrom, Jeffrey Blanchard, Tanja Woyke

This PDF file includes:

Supplementary Tables 1-7
Supplementary Figures 1-9
Supplementary References

Supplementary Table 1. Assembly summary of soil giant viruses. Genomes are visually compared in Supplementary Fig. 2. * These viral genomes were excluded from further analysis due to low genome completeness based on presence/absence of conserved NCVOGs [1] (Supplementary Fig. 1). **Partial genomes were deposited at https://bitbucket.org/berkeleylab/forestsoil-gv

Name	Assembly Size [bp]	Genes	GC [%]	Contigs (>2 kb)	N50 [bp]	Accession (NCBI)
3300020774-4*	376,227	440	30	47	12,269	**
3300020905-1*	231,229	244	37	31	8,947	**
3300020918-1*	597,079	568	36	55	13,574	**
Barrevirus BAV1	604,913	587	32	44	20,887	MK071998-MK072041
Dasosvirus DSV1	301,586	304	31	24	35,562	MK072042-MK072065
Edafosvirus EDV1	1,255,552	1,311	28	66	35,172	MK072066-MK072131
Faunusvirus FNV1	812,113	838	31	67	18,668	MK072132-MK072198
Gaeavirus GAV1	549,019	512	32	44	18,526	MK072199-MK072242
Harvfovirus HAV1	1,553,620	1,596	36	89	23,970	MK072243-MK072331
Homavirus HOV1	456,650	451	29	51	11,209	MK072332-MK072382
Hyperionvirus HYV1	2,377,404	2,494	37	54	69,817	MK072383-MK072436
Satyrvirus SAV1	904,917	929	31	52	27,026	MK072437-MK072488
Solivirus SOV1	276,500	282	37	9	70,714	MK072489-MK072497
Solumvirus SMV1	316,086	279	35	9	47,043	MK072498-MK072506
Sylvanvirus SYV1	940,362	853	36	45	34,512	MK072507-MK072551
Terrestrivirus TEV1	1,790,239	1,652	29	19	251,584	MK071979-MK071997

Supplementary Table 2. Naming considerations. Proposed names and etymology for the thirteen novel giant viruses with the most complete genomes. * Not affiliated with existing viral lineages on the genus, subfamily and family level. Naming of the here discovered metagenomic viruses is in accordance with the Consensus Statement on “Viral Taxonomy in the Age of Metagenomics” [2].

Proposed Name	Viral Lineage	Etymology
**Barrevirus**	Klosneuvirinae	N. L. n. Barre- referring to the Barre Woods long-term experimental warming site from which the soil samples were collected.
**Dasosvirus**	Klosneuvirinae	Gr. n. Dasos-, forest
**Faunusvirus**	Mimiviridae	L. n. Faunus- referring to the Roman horned god of the forest, plains, and fields.
**Edafosvirus**	Klosneuvirinae	Gr. n. Edafos-, terrain
**Gaeavirus**	Klosneuvirinae	Gr. n. Gaea-, referring to the ancient Greek goddess representing the personification of earth
**Harvfovirus**	Klosneuvirinae	N. L. n. Harv- referring to Harvard, -fo- referring to forest, denoting the site from which the soil samples were collected.
**Homavirus**	Klosneuvirinae	N. L. n. Homa-, referring to the tree of life
**Hyperionvirus**	Klosneuvirinae	G. n. Hyperion-, referring to the world's tallest known living tree..
**Satyrvirus**	Megamimivirinae	Gr. n. Satyr- referring to the ancient Greek deities of the woods and mountains.
**Solivirus**	Megavirales*	L. adj. Soli-, soil
**Solumvirus**	Megavirales*	L. adj. Solum-, soil
**Sylvanvirus**	Megavirales*	L. n. Sylva- , timber
**Terrestrivirus**	Klosneuvirinae	L. n. Terrestri-, from latin terrestrius, earth

Supplementary Table 3. Metadata summary of samples used for bulk- and mini-metagenomics and metatranscriptomics. *These viral genomes were excluded from further analysis due to low genome completeness.

Metagenome Accession (IMG)	GOLD ID	Treatment	Soil Type	Giant Virus Identified
3300020974	Ga0206957	Heated	Organic	Sylvanvirus
3300020918	Ga0207107	Heated	Mineral	3300020918-1*
3300020905	Ga0207100	Heated	Mineral	3300020905-1*
3300020904	Ga0207088	Heated	Mineral	Homavirus
3300020831	Ga0207143	Heated	Mineral	Dasosvirus
3300020823	Ga0207075	Heated	Mineral	Solumvirus
3300020795	Ga0206962	Heated	Organic	Barrevirus
3300020774	Ga0206948	Heated	Organic	3300020774-4*
3300020767	Ga0206992	Control	Mineral	Satyrvirus
3300020765	Ga0207047	Control	Mineral	Harvfovirus
3300020757	Ga0206990	Control	Mineral	Hyperionvirus
3300020744	Ga0207027	Control	Mineral	Gaeavirus
3300020659	Ga0206851	Control	Organic	Solivirus
3300020651	Ga0206911	Heated	Organic	Edafosvirus
3300020631	Ga0206834	Control	Organic	Terrestrivirus
3300020581	Ga0210399	Control	Mineral	Faunusvirus

Supplementary Table 4. Regions in genome assemblies with low read coverage. Positions (start, stop) of regions (300 bp) with an average read coverage of 3 or less are indicated and the average contig read coverage. Contig edges (500 bp) were not taken into account.

Contig	Contig size [bp]	Start	Stop	Coverage	Contig coverage
Barrevirus_14	15494	2101	2400	3	20
Barrevirus_19	11371	6301	6600	2	12
Barrevirus_23	9093	6301	6600	3	10
Dasosvirus_14	4840	901	1200	3	7
Dasosvirus_2	38788	17701	18000	2	26
Dasosvirus_22	2767	601	900	3	5
Dasosvirus_6	14703	3001	3300	3	17
Edafosvirus_24	14887	12901	13200	2	15
Edafosvirus_25	14139	2401	2700	3	13
Edafosvirus_41	6708	601	900	3	10
Edafosvirus_51	3824	1201	1500	3	9
Edafosvirus_56	3532	2701	3000	3	22
Edafosvirus_6	51907	50101	50400	3	82
Edafosvirus_61	3038	1501	1800	3	7
Edafosvirus_8	38472	13801	14100	3	22
Gaeavirus_16	11195	9301	9600	3	41
Harvfovirus_5	50546	45001	45300	3	144
Harvfovirus_65	7756	3601	3900	3	12
Harvfovirus_66	7588	6301	6600	3	38
Homavirus_16	9845	6301	6600	2	14
Homavirus_39	4225	2401	2700	3	9
Hyperionvirus_20	44408	41401	41700	3	348
Hyperionvirus_32	24307	1501	1800	3	26
Hyperionvirus_34	22059	19801	20100	3	26
Satyrvirus_25	13648	9601	9900	3	21
Satyrvirus_30	11419	2401	2700	3	14
Satyrvirus_37	8727	5401	5700	1	12
Solivirus_4	38098	37201	37500	3	56
Sylvanvirus_14	25196	24301	24600	3	28
Sylvanvirus_33	7479	6001	6300	3	61
Sylvanvirus_4	46678	13501	13800	2	24
Terrestrivirus_3	271924	61201	61500	3	178

Supplementary Table 5. Recovery of soil giant virus genes in metatranscriptomes. Giant virus genes which could be assembled from the metatranscriptomes (MTT).

Gene	MTT assembled	Average fold coverage	Identity [%]	Covered [bp]	Product
3300020905-1_1_49	Ga0242656_1223623	3.2172	99.6	295	hypothetical protein
3300020905-1_1_103	Ga0242662_10892879	1.8634	100	322	AEX62687.1 hypothetical protein mv_L482 [Moumouvirus Monve]
3300020918-1_1_17	Ga0242662_10127587	1.9281	100	751	AGD92382.1 capsid protein 1 [Megavirus lba]
3300020918-1_1_18	Ga0242662_10190859	3.2172	100	129	hypothetical protein
3300020918-1_1_196	Ga0242662_10164121	1.9838	99.7	681	AGF85257.1 hypothetical protein glt_00448 [Moumouvirus goulette]
Barrevirus_7_8	Ga0242670_1014965	2.0751	100	165	hypothetical protein
Barrevirus_7_10	Ga0242670_1286930	3.2226	100	125	hypothetical protein
Solumvirus_3_10	Ga0242655_10912936	1.7335	100	319	hypothetical protein
Solumvirus_5_25	Ga0242678_1214968	10.9477	100	215	hypothetical protein
Terrestrivirus_4_114	Ga0242668_1460315	1.142	97.8	187	hypothetical protein

Supplementary Table 6. Taxonomic classification of eukaryotic 18S rRNA gene sequences extracted from bulk metagenomes. From mini- and bulk metagenomes, 18S rRNA gene sequences were identified using Infernal cmsearch with a covariance model of the eukaryotic 18S ribosomal RNA (RF01960) [3,4]. Hits were extracted and searched against the SILVA database (v132)[5] using blastn [6]. The table shows only hits to protists with an alignment length of at least 1200 bp. In addition to hits against Apicomplexa, Ciliophara and Cercozao there were 63 hits against fungi and 25 hits against metazoa. No eukaryotic 18S ribosomal RNA gene sequences of any length were detected in the mini-metagenomes which contained the giant viruses.

Contigname (IMG)	Position	Best hit (SILVA)	Identity [%]	Length [bp]	Taxonomy of best hit
Ga0210397_10010074	1022_2808	GQ330636.1.1435	99.168	1443	SAR;Alveolata;Apicomplexa;Conoidasida;Gregarinasina;Archigregarinorida;Selenidium;uncultured Coccidia
Ga0210387_10010255	1474_3260	GQ330636.1.1435	99.168	1443	SAR;Alveolata;Apicomplexa;Conoidasida;Gregarinasina;Archigregarinorida;Selenidium;uncultured Coccidia
Ga0210385_10003826	1014_2800	GQ330636.1.1435	99.168	1443	SAR;Alveolata;Apicomplexa;Conoidasida;Gregarinasina;Archigregarinorida;Selenidium;uncultured Coccidia
Ga0210387_10036899	683_2458	EF024928.1.1761	99.091	1761	SAR;Alveolata;Ciliophora;Intramacronucleata;Spirotrichea;Hypotrichia;Gonostomum;uncultured Oxytrichidae
Ga0210387_10034108	1_1294	FPLS01020493.8.1436	97.98	1287	SAR;Rhizaria;Cercozoa;Thecofilosea;uncultured;metagenome
Ga0210408_10074274	351_2127	EF024338.1.1850	96.82	1761	SAR;Alveolata;Apicomplexa;Conoidasida;Gregarinasina;Eugregarinorida;uncultured Eimeriidae
Ga0210404_10018451	299_2075	EF024338.1.1850	96.82	1761	SAR;Alveolata;Apicomplexa;Conoidasida;Gregarinasina;Eugregarinorida;uncultured Eimeriidae
Ga0210385_10009821	1721_3526	AF411281.1.1803	94.539	1813	SAR;Rhizaria;Cercozoa;Imbricatea;Spongomonadida;Spongomonas;Spongomonas minima
Ga0210393_10102083	991_2284	FJ865354.1.1749	85.453	1313	SAR;Alveolata;Apicomplexa;Conoidasida;Gregarinasina;Eugregarinorida;Psychodiella;Psychodiella chagasi
Ga0210387_10109362	1027_2321	FJ865354.1.1749	84.932	1314	SAR;Alveolata;Apicomplexa;Conoidasida;Gregarinasina;Eugregarinorida;Psychodiella;Psychodiella chagasi

Supplementary Table 7. Taxonomic classification non viral metagenome assembled genomes (MAGs) in the minimetagenomes. Taxonomy of MAGs was assigned with genome taxonomy database (GTDB) classifier (https://github.com/Ecogenomics/GTDBTk).

Accession (IMG)	Taxonomy (GTDB)
3300020651-2	d__Bacteria;p__Bacteroidetes;c__Bacteroidia;o__Chitinophagales;f__LD1
3300020651-3	d__Bacteria;p__Verrucomicrobia;c__Verrucomicrobiae;o__Pedosphaerales;f__Pedosphaeraceae
3300020651-4	d__Bacteria;p__Elusimicrobia;c__Elusimicrobia;o__2-01-FULL-59-12;f__2-01-FULL-59-12;g__2-01-FULL-59-12
3300020651-5	d__Bacteria;p__Oligoflexaeota
3300020651-6	d__Bacteria;p__Acidobacteria;c__Acidobacteriia;o__Acidobacteriales;f__Acidobacteriaceae;g__KBS-89
3300020659-1	d__Bacteria;p__Bacteroidetes;c__Bacteroidia;o__AKYH767;f__b-17BO
3300020659-2	d__Bacteria;p__Verrucomicrobia;c__Verrucomicrobiae;o__Chthoniobacterales
3300020659-3	d__Bacteria;p__Bacteroidetes;c__Bacteroidia;o__Chitinophagales;f__Chitinophagaceae;g__UBA8621
3300020659-5	d__Bacteria;p__Acidobacteria;c__Acidobacteriia;o__Acidobacteriales;f__Acidobacteriaceae
3300020659-6	d__Bacteria;p__Bacteroidetes;c__Bacteroidia;o__Chitinophagales;f__Chitinophagaceae;g__UBA8621
3300020744-2	d__Bacteria;p__Bacteroidetes;c__Bacteroidia;o__Chitinophagales;f__Chitinophagaceae;g__Ferruginibacter
3300020744-3	d__Bacteria;p__Proteobacteria;c__Gammaproteobacteria;o__UBA5158;f__UBA5158
3300020744-4	d__Bacteria;p__Bacteroidetes;c__Bacteroidia;o__AKYH767
3300020744-5	d__Bacteria;p__Bacteroidetes;c__Bacteroidia;o__Sphingobacteriales;f__Sphingobacteriaceae;g__Mucilaginibacter
3300020757-1	d__Bacteria;p__Proteobacteria;c__Gammaproteobacteria;o__UBA5158;f__UBA5158;g__2-12-FULL-45-12
3300020757-2	d__Bacteria;p__Bacteroidetes;c__Bacteroidia;o__AKYH767;f__b-17BO
3300020757-3	d__Bacteria;p__Acidobacteria;c__Acidobacteriia;o__Acidobacteriales
3300020757-4	d__Bacteria;p__Bacteroidetes
3300020765-2	d__Bacteria;p__Acidobacteria;c__Acidobacteriia;o__Acidobacteriales
3300020765-3	d__Bacteria;p__Bacteroidetes;c__Bacteroidia;o__Chitinophagales;f__Chitinophagaceae
3300020765-4	d__Bacteria;p__Bacteroidetes;c__Bacteroidia;o__Sphingobacteriales;f__Sphingobacteriaceae;g__Mucilaginibacter
3300020765-5	d__Bacteria;p__Proteobacteria;c__Gammaproteobacteria;o__Betaproteobacteriales;f__Burkholderiaceae
3300020767-1	d__Bacteria;p__Acidobacteria;c__Acidobacteriia;o__20CM-2-55-15
3300020767-2	d__Bacteria;p__Proteobacteria;c__Alphaproteobacteria
3300020767-3	d__Bacteria;p__Proteobacteria;c__Gammaproteobacteria;o__Betaproteobacteriales;f__Burkholderiaceae
3300020767-4	d__Bacteria;p__Acidobacteria;c__Acidobacteriia;o__Acidobacteriales;f__Acidobacteriaceae;g__Silvibacterium
3300020767-6	d__Bacteria;p__Cyanobacteria;c__Melainabacteria;o__Obscuribacterales;f__Obscuribacteraceae
3300020774-1	d__Bacteria;p__Dependentiae;c__Babeliae;o__Babeliales;f__Vermiphilaceae
3300020774-2	d__Bacteria;p__Proteobacteria;c__Alphaproteobacteria;o__Rickettsiales;f__Midichloriaceae
3300020774-3	d__Bacteria;p__Proteobacteria;c__Alphaproteobacteria;o__Micavibrionales;f__Micavibrionaceae
3300020774-5	d__Bacteria;p__Verrucomicrobia;c__Verrucomicrobiae;o__Pedosphaerales;f__Pedosphaeraceae
3300020774-6	d__Bacteria;p__Armatimonadetes;c__Fimbriimonadia;o__Fimbriimonadales;f__Fimbriimonadaceae;g__55-13
3300020774-7	d__Bacteria;p__Proteobacteria;c__Alphaproteobacteria;o__Tistrellales;f__Tistrellaceae
3300020795-1	d__Bacteria;p__Proteobacteria;c__Alphaproteobacteria;o__Micavibrionales;f__UBA1247
3300020795-2	d__Bacteria;p__Bacteroidetes;c__Bacteroidia;o__AKYH767;f__b-17BO
3300020795-3	d__Bacteria;p__Bacteroidetes;c__Bacteroidia
3300020795-4	d__Bacteria;p__Proteobacteria;c__Gammaproteobacteria;o__UBA5158;f__UBA5158
3300020795-6	d__Bacteria;p__Acidobacteria;c__Acidobacteriia;o__Acidobacteriales;f__Acidobacteriaceae
3300020795-7	d__Bacteria;p__Proteobacteria;c__Alphaproteobacteria
3300020823-1	d__Bacteria;p__Dependentiae;c__Babeliae;o__Babeliales;f__RVW-14
3300020823-2	d__Bacteria;p__Proteobacteria;c__Gammaproteobacteria;o__HT99;f__HT99
3300020831-1	d__Bacteria;p__Proteobacteria;c__Gammaproteobacteria;o__UBA5158;f__UBA5158
3300020831-2	d__Bacteria;p__Proteobacteria;c__Gammaproteobacteria;o__UBA5158;f__UBA5158
3300020831-3	d__Bacteria;p__Verrucomicrobia;c__Chlamydiia;o__Chlamydiales;f__Simkaniaceae
3300020904-1	d__Bacteria;p__Acidobacteria;c__Acidobacteriia;o__Acidobacteriales;f__Koribacteraceae
3300020904-2	d__Bacteria;p__Verrucomicrobia;c__Chlamydiia;o__Chlamydiales
3300020904-3	d__Bacteria;p__Bacteroidetes;c__Bacteroidia;o__Sphingobacteriales;f__Sphingobacteriaceae;g__Mucilaginibacter
3300020904-4	d__Bacteria;p__Bacteroidetes
3300020904-5	d__Bacteria;p__Bacteroidetes;c__Bacteroidia;o__Chitinophagales;f__Chitinophagaceae;g__Ferruginibacter
3300020904-6	d__Bacteria;p__Proteobacteria;c__Gammaproteobacteria;o__HT99
3300020904-7	d__Bacteria;p__Dependentiae;c__Babeliae;o__Babeliales;f__RVW-14;g__UASB124
3300020905-2	d__Bacteria;p__Elusimicrobia;c__Elusimicrobia;o__2-01-FULL-59-12
3300020905-3	d__Bacteria;p__Bacteroidetes
3300020905-4	d__Bacteria;p__Acidobacteria;c__Acidobacteriia;o__Acidobacteriales;f__Koribacteraceae
3300020918-2	d__Bacteria;p__Acidobacteria;c__Acidobacteriia;o__UBA7541;f__UBA7541
3300020918-3	d__Bacteria;p__Proteobacteria;c__Gammaproteobacteria;o__HT99
3300020918-4	d__Bacteria;p__Proteobacteria;c__Alphaproteobacteria
3300020918-5	d__Bacteria;p__Verrucomicrobia;c__Verrucomicrobiae
3300020974-1	d__Bacteria;p__Proteobacteria;c__Alphaproteobacteria
3300020974-2	d__Bacteria;p__Proteobacteria;c__Alphaproteobacteria
3300020974-3	d__Bacteria;p__Omnitrophica;c__koll11;o__UBA10015
3300020974-5	d__Bacteria;p__Bacteroidetes;c__Bacteroidia;o__AKYH767;f__b-17BO

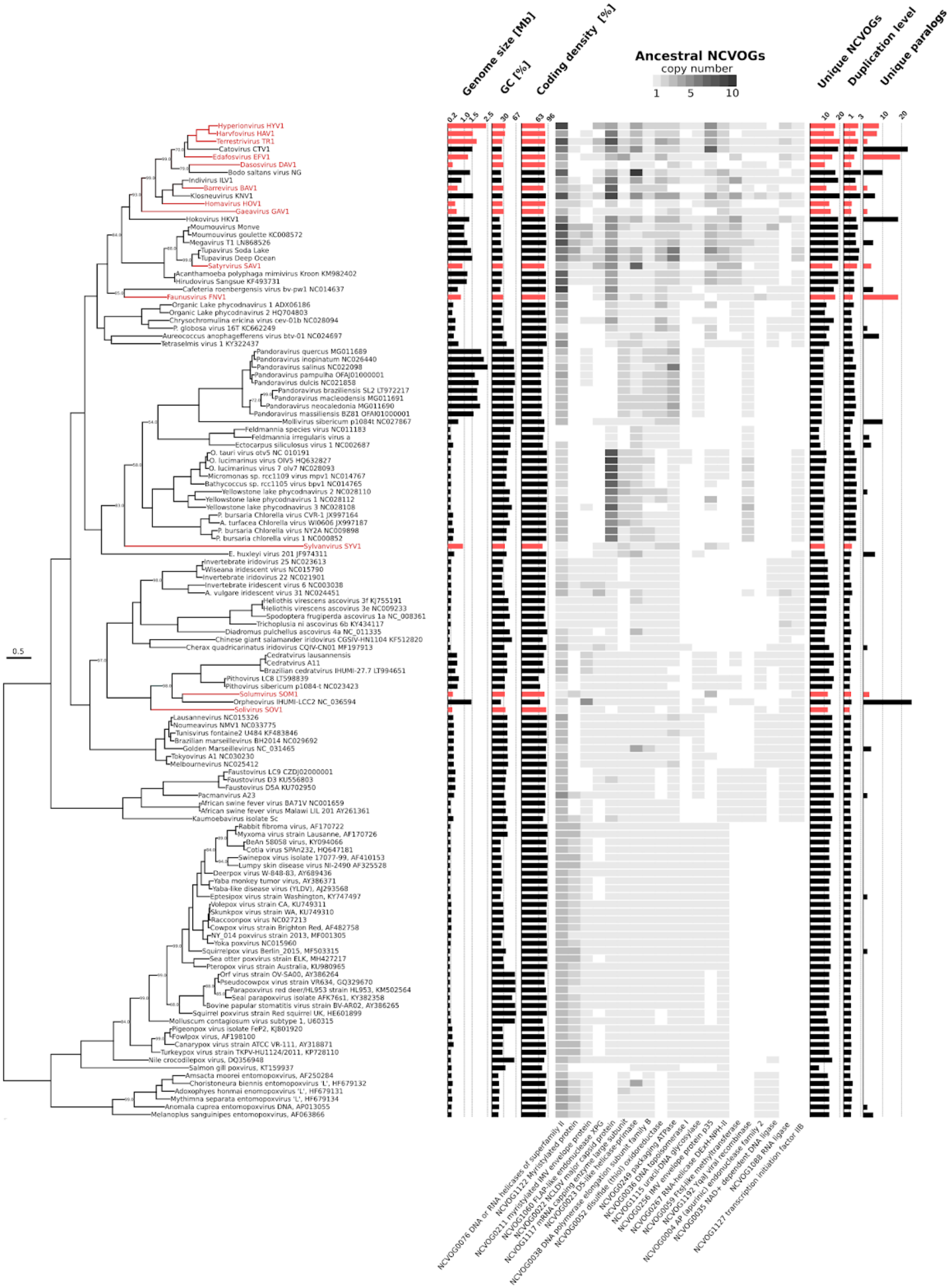

Genome size [Mb]
GC [%]
Coding density [%]
Ancestral NCVOGs
copy number
1
5
10
Unique NCVOGs
Duplication level
Unique paralogs
0.5

Supplementary Fig. 1. Genome features and genome completeness estimate of novel soil giant viruses. Nucleocytoplasmic large DNA virus (NCLDV) maximum likelihood tree (Fig. 2), with novel giant virus genomes described in this study highlighted in red. Branches are collapsed if support was low (<50), support values are not indicated in case of full support (100). The tree is rooted at the *Poxviridae*. As measure for genome completeness and contamination the copy number of 20 ancestral NCVOGs [1], total number of unique ancestral NCVOGs and their duplication level (total number of ancestral NCVOGs divided by total number of unique ancestral NCVOGs) are indicated. Presence/absence and copy numbers of ancestral NCVOGs featured in the new genomes are similar to closely related previously published genomes. The only outlier Bodo saltans virus with 20 copies of NCVOG0023 is displayed in the heatmap as 10. More details on copy number of ancestral NCVOGs provided in Supplementary Fig. 4. Bars on the very right indicate the total number of genes in paralogous gene families unique to the respective viral genomes. The scale bar represents substitutions per site.

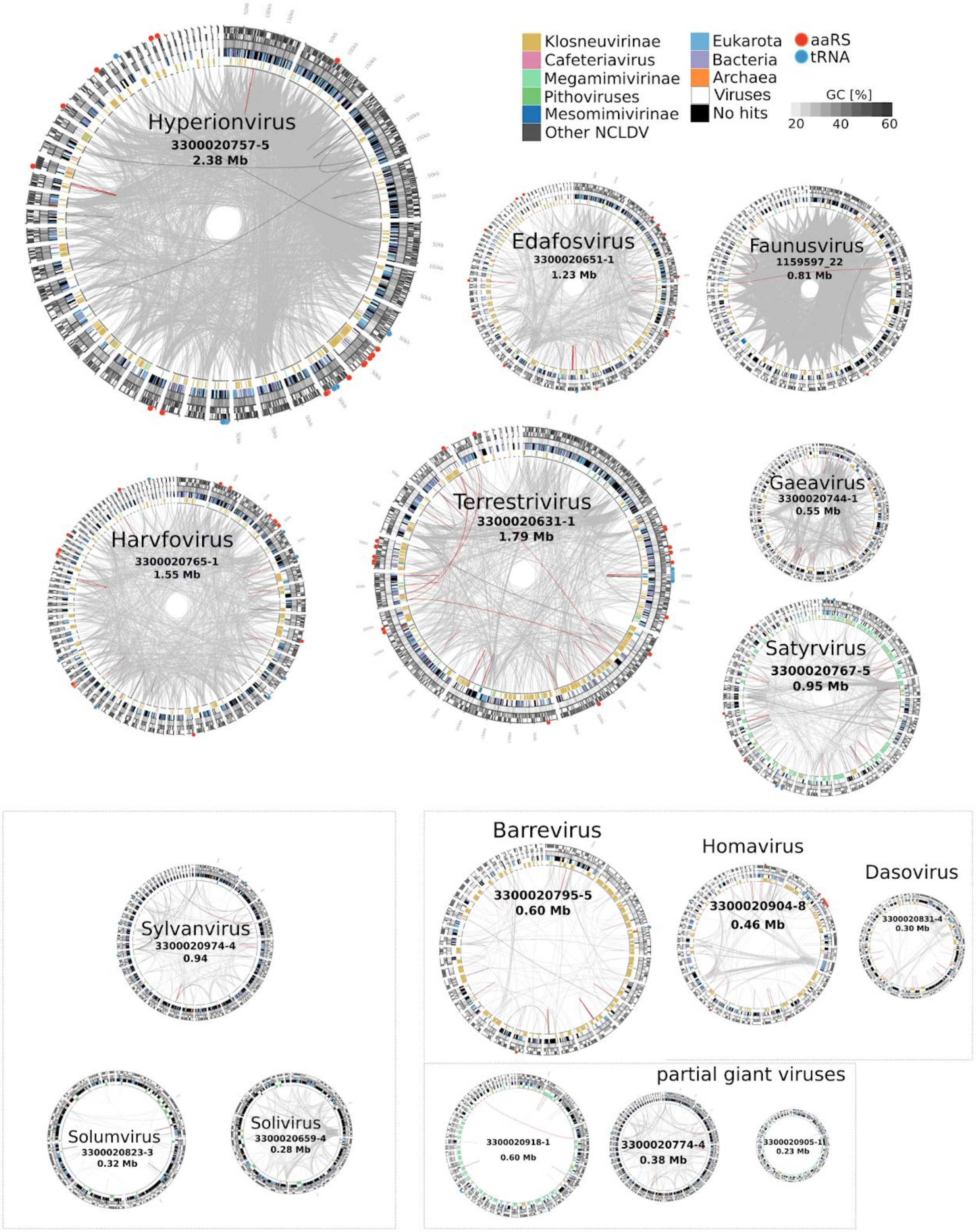

Klosneuvirinae
Cafeteriavirus
Megamimivirinae
Pithoviruses
Mesomimivirinae
Other NCLDV
Eukarota
Bacteria
Archaea
Viruses
No hits
aaRS
tRNA
GC [%]
20
40
60
Hyperionvirus
3300020757-5
2.38 Mb
Edafosvirus
3300020651-1
1.23 Mb
Faunusvirus
1159597_22
0.81 Mb
Harvfovirus
3300020765-1
1.55 Mb
Terrestrivirus
3300020631-1
1.79 Mb
Gaeavirus
3300020744-1
0.55 Mb
Satyrvirus
3300020767-5
0.95 Mb
Sylvanvirus
3300020974-4
0.94
Barrevirus
3300020795-5
0.60 Mb
Homavirus
3300020904-8
0.46 Mb
Dasovirus
3300020831-4
0.30 Mb
Solumvirus
3300020823-3
0.32 Mb
Solivirus
3300020659-4
0.28 Mb
partial giant viruses
3300020918-1
0.60 Mb
3300020774-4
0.38 Mb
3300020905-1
0.23 Mb

Supplementary Fig. 2. Circular representation of novel soil giant virus genomes. Each plot shows from outside to inside: Filled circles depict location of encoded tRNAs and aminoacyl-tRNA synthetases (aaRS). The second ring displays positions of genes (gray) either on the minus or the plus strand. The next track illustrates GC content in shades of gray. The fourth track shows color-coded origin of proteins with best blastp hits (evalue 1e-5) to cellular homologs, best hits against viral proteins are indicated in white and if possible, further broken down based on their taxonomic origin color-coded on the most inner track. Finally, lines in the middle of the plot connect paralogs (gray) and nearly identical repeats (orange).

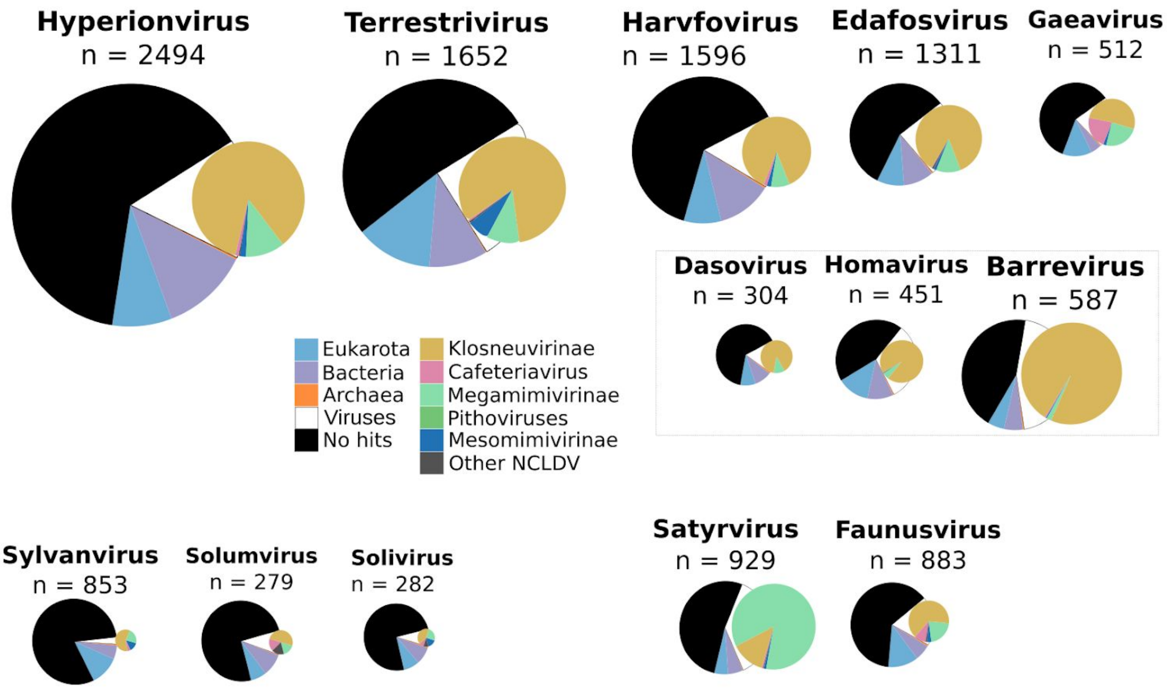

Supplementary Fig. 3. Taxonomic breakdown of genes encoded by novel soil giant viruses. Pie charts summarizes percentage of genes with and without cellular homologs, which are further broken down based on taxonomic affiliation of best blastp hits (evalue 1e-5).

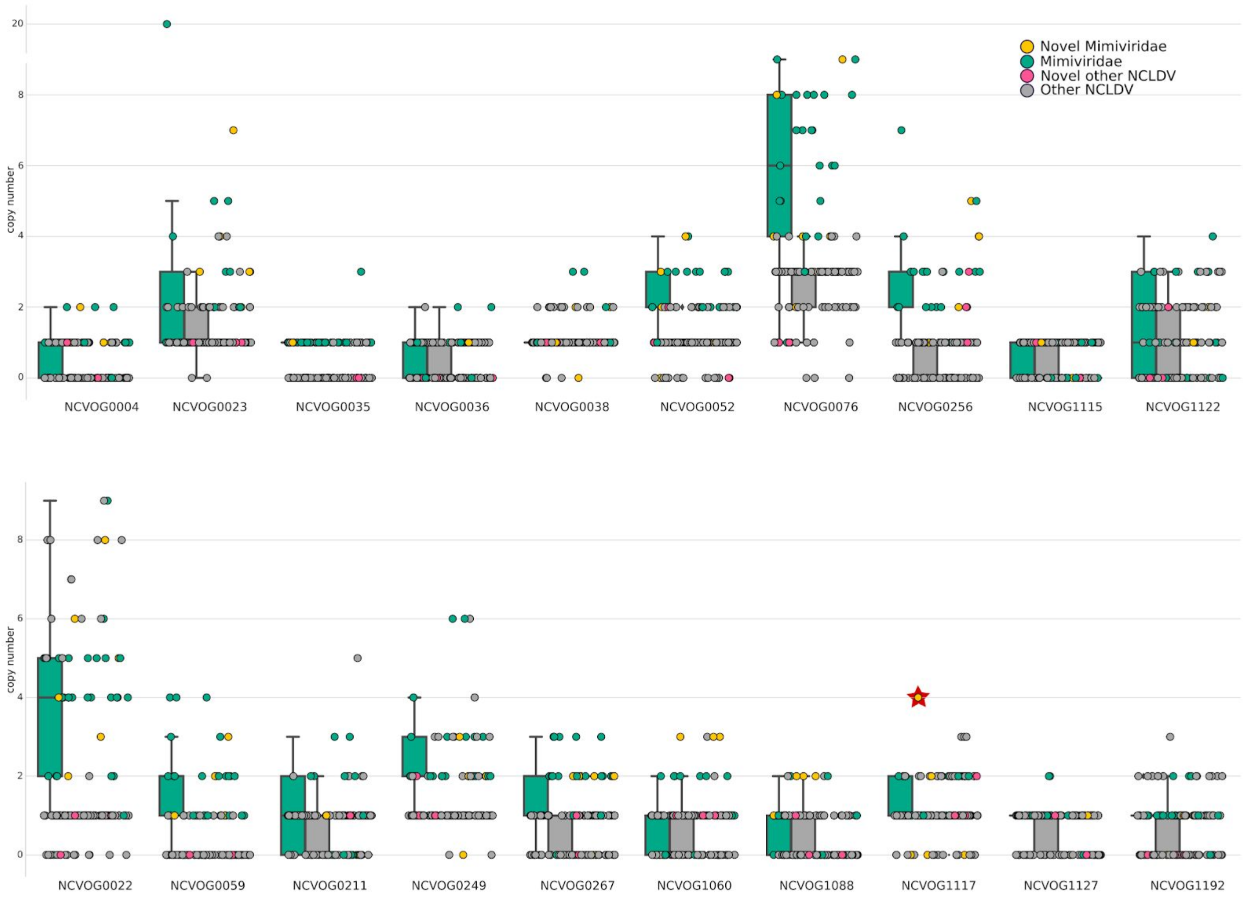

Supplementary Fig. 4 Duplication level of ancestral NCVOGs. As measure for genome completeness and contamination copy numbers for each of the 20 ancestral NCVOGs [1] are indicated. Copy numbers of ancestral NCVOGs in the new soil giant virus genomes are similar to other previously published NCLDV genomes. The only exception is NCVOG1117 (mRNA capping enzyme large subunit), for which *Ca.* Edafosvirus has an additional copy compared to previously published NCLDV genomes. Center lines of boxplots represent the median, bounds of boxes the lower and upper quartile, whiskers extend to points that lie within 1.5 interquartile range of the lower and upper quartile.

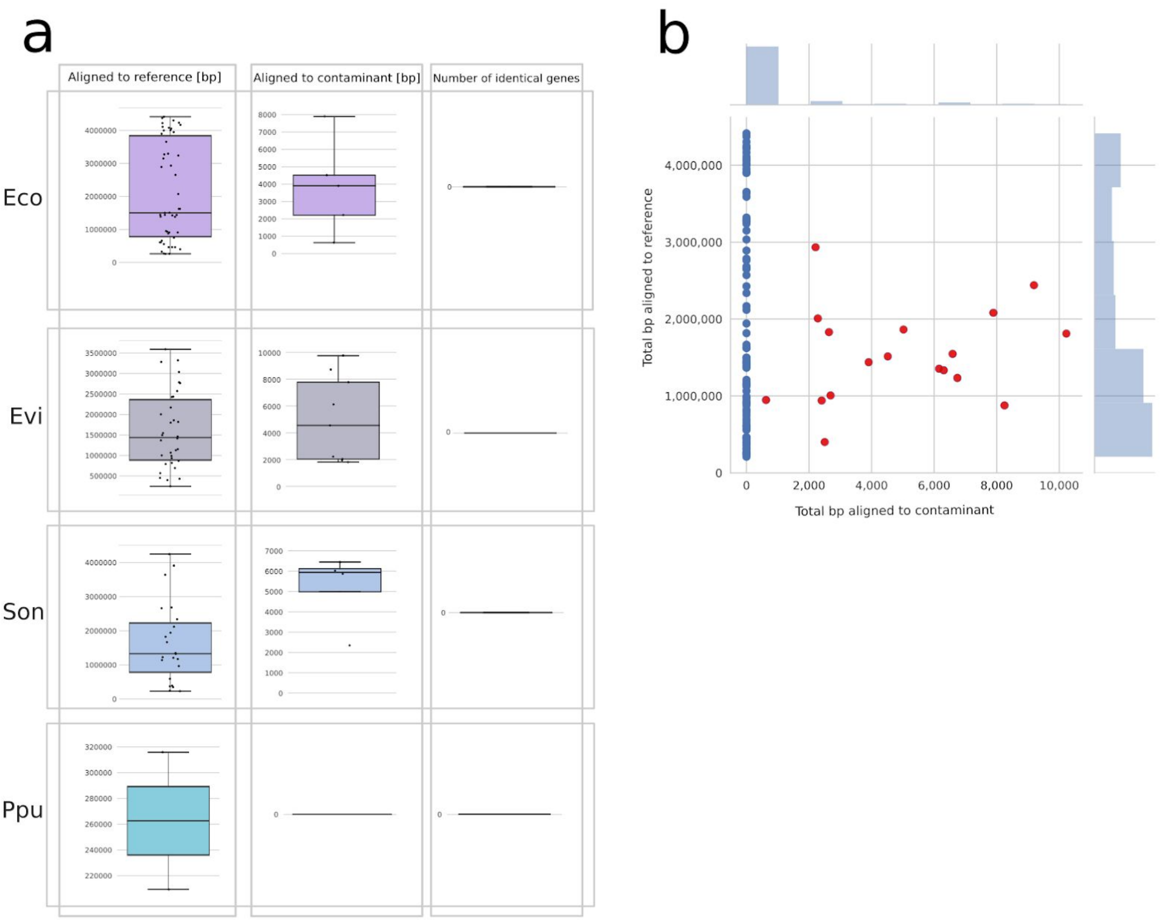

Supplementary Fig. 5. Benchmarking of the mini-metagenomics approach and metagenomic binning. Mini-metagenomics benchmarking based on randomly sorting of 10 cells from a bacterial mock community consisting of five different bacterial isolates, Eco: *Escherichia coli* K12, Evi: *Echinicola vietnamensis* DSM 17526, Son: *Shewanella oneidensis* MR-1, Ppu: *Pseudomonas putida* F1; Mru *Meiothermus ruber*. Mru was not recovered with our approach. **a** Box plots indicating number of nucleotides which could be aligned to reference genomes and nucleotides in the respective MAG which mapped to another reference organism ("contaminant") present in the same mini-metagenome. Furthermore, the number of identical genes found in each MAG (indicator for chimeric sequence duplication) is indicated and the average number of duplicated (100% identity) genes per MAG. **b** An alternative view of the number of nucleotides in each MAG which could be aligned to the correct reference genome or to contaminants. In summary, the benchmarking experiment showed only a very low rate of binning-derived contamination: 1-2 contaminant contigs in 18 out of 109 total bins, affecting less than 2% of sequence information contained in the respective MAG. More importantly, the contamination was derived from wrong binning and there was not a single case in which MDA gave rise to true chimeric sequences (sequences patched together from different microbes on a single contig). Center lines of boxplots represent the median, bounds of boxes the lower and upper quartile, whiskers extend to points that lie within 1.5 interquartile range of the lower and upper quartile.

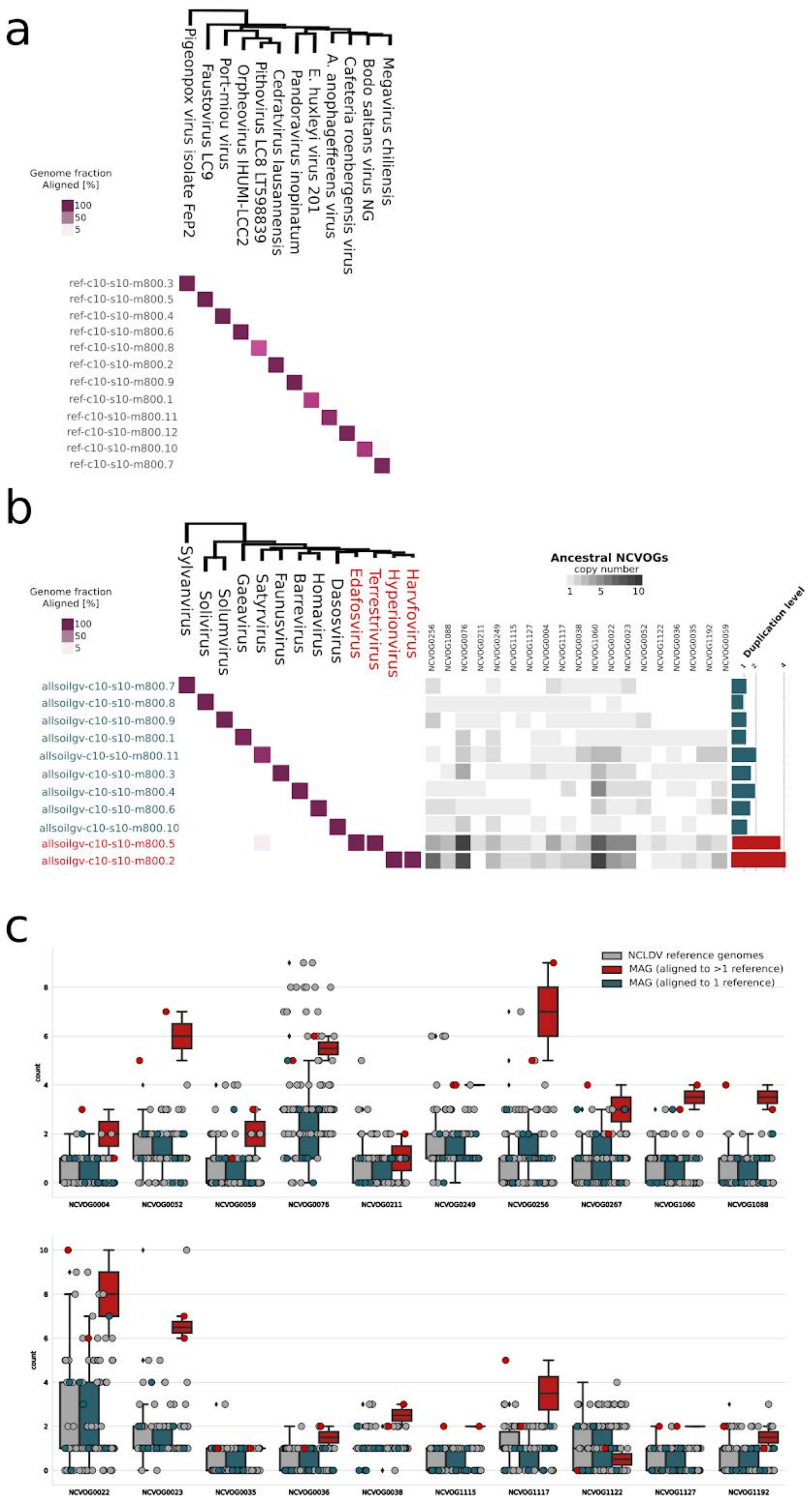
a
Megavirus chiliensis
Bodo saltans virus NG
Cafeteria roenbergensis virus
A. anophagefferens virus
E. huxleyi virus 201
Pandoravirus inopinatum
Cedratvirus lausannensis
Pithovirus LC8 LT598839
Orpheovirus IHUMI-LCC2
Port-miou virus
Faustovirus LC9
Pigeonpox virus isolate FeP2
Genome fraction Aligned [%]
100
50
5
ref-c10-s10-m800.3
ref-c10-s10-m800.5
ref-c10-s10-m800.4
ref-c10-s10-m800.6
ref-c10-s10-m800.8
ref-c10-s10-m800.2
ref-c10-s10-m800.9
ref-c10-s10-m800.1
ref-c10-s10-m800.11
ref-c10-s10-m800.12
ref-c10-s10-m800.10
ref-c10-s10-m800.7
b
Sylvanvirus
Solivirus
Solumvirus
Gaeavirus
Satyrvirus
Faunusvirus
Barrevirus
Homavirus
Dasosvirus
Edafosvirus
Terrestrivirus
Hyperionvirus
Harvfovirus
Ancestral NCVOGs
copy number
1
5
10
Duplication level
allsoilgv-c10-s10-m800.7
allsoilgv-c10-s10-m800.8
allsoilgv-c10-s10-m800.9
allsoilgv-c10-s10-m800.1
allsoilgv-c10-s10-m800.11
allsoilgv-c10-s10-m800.3
allsoilgv-c10-s10-m800.4
allsoilgv-c10-s10-m800.6
allsoilgv-c10-s10-m800.10
allsoilgv-c10-s10-m800.5
allsoilgv-c10-s10-m800.2
c
NCLDV reference genomes
MAG (aligned to >1 reference)
MAG (aligned to 1 reference)
count
NCVOG0004
NCVOG0052
NCVOG0059
NCVOG0076
NCVOG0211
NCVOG0249
NCVOG0256
NCVOG0267
NCVOG1060
NCVOG1088
NCVOG0022
NCVOG0023
NCVOG0035
NCVOG0036
NCVOG0038
NCVOG1115
NCVOG1117
NCVOG1122
NCVOG1127
NCVOG1192

Supplementary Fig. 6 Simulation of metagenomic binning. a Binning of a simulated mock community consisting of 12 giant viruses, each a representative of a subfamily or family in the *Megavirales*. Alignment fraction of in total 12 MAGs to reference genomes is shown. Only one MAG (Bodo saltans virus) was affected by contamination, containing a single contaminant contig (from Megavirus chilensis, size < 20kb, ~1.5% of the total MAG size). **b** Binning of mock community simulated from the 13 most complete soil giant virus genomes discovered in this study. Two out of 11 MAGs were chimeric, both consisting of more than one closely related virus (highlighted in red). As measure for genome completeness and contamination the copy numbers of 20 ancestral NCVOGs [1] and their duplication level (total number of ancestral NCVOGs divided by total number of unique ancestral NCVOGs) are indicated. Chimeric MAGs can be detected using this approach. **c** A detailed view of copy numbers for each of the 20 ancestral NCVOGs for simulated MAGs (**b**) compared to other *Megavirales* genomes. Chimeric MAGs can be detected using this approach. Center lines of boxplots represent the median, bounds of boxes the lower and upper quartile, whiskers extend to points that lie within 1.5 interquartile range of the lower and upper quartile.

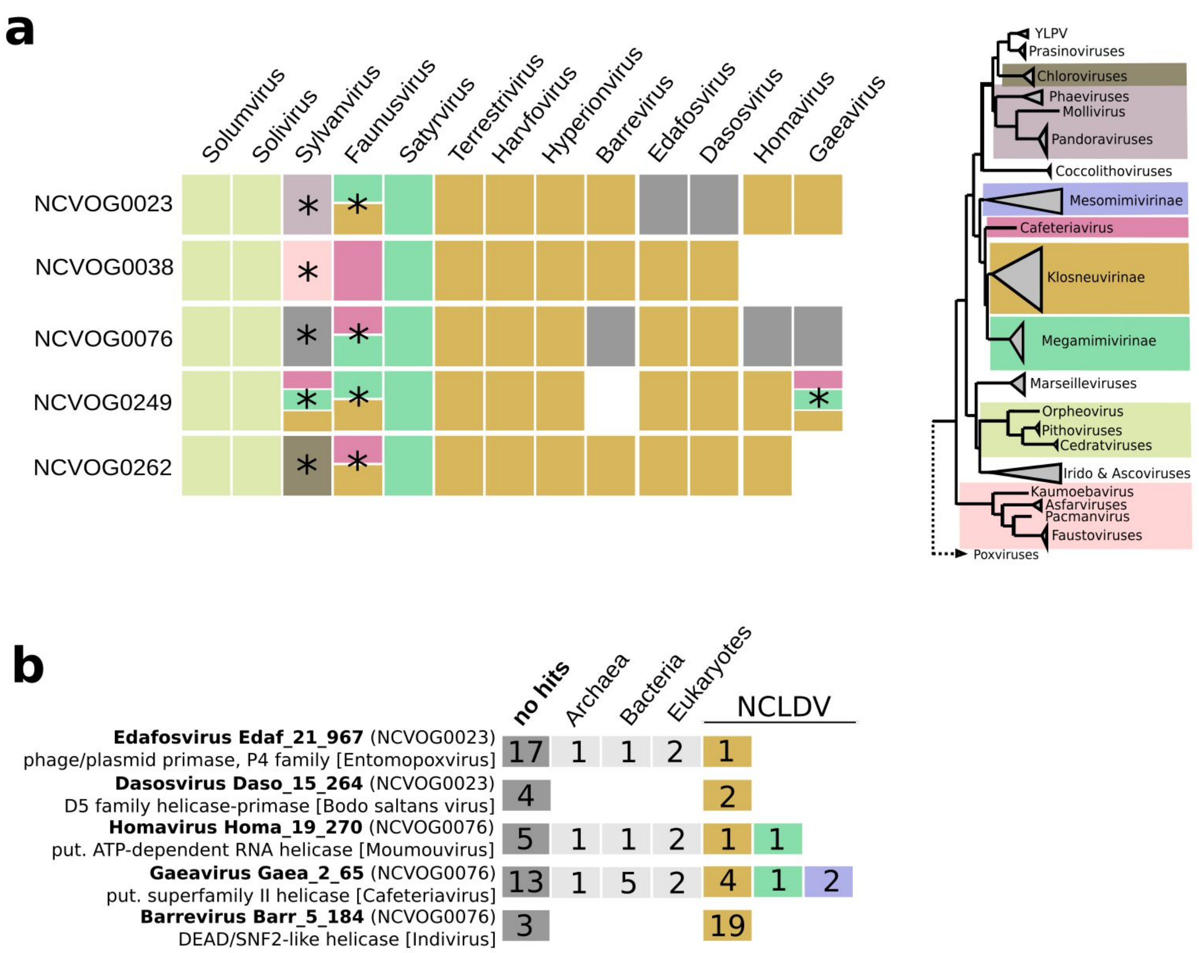

Supplementary Fig. 7. Phylogenomic placement of novel viral lineages based on single protein phylogenies. a Summary of phylogenetic placement of each of the five core NCVOGs selected for the concatenated species tree (Fig. 2, Supplementary Fig. 1, Supplementary Fig. 7): DNA polymerase elongation subunit family B (NCVOG0038), D5-like helicase-primase (NCVOG0023), packaging ATPase (NCVOG0249), Poxvirus Late Transcription Factor VLTF3-like (NCVOG0262), and DNA or RNA helicases of superfamily II (NCVOG0076). Phylogenetic affiliation to reference lineage is color coded, with lineage-specific colors indicated in the schematic representation of the NCLDV species tree shown in the right panel. * indicates basal position of the respective gene, e.g. the selected copy of NCVOG0249 in *Ca.* Sylvanvirus groups basal to its counterparts in *Cafeteriavirus*, *Megamimivirinae* and *Klosneuviriniae*. Grey color indicates a potential gene replacement or divergence after duplication, i.e. the respective gene was affiliated with a different NCLDV lineage than in the species tree (Fig. 2, Supplementary Fig. 1). **b** Detailed inspection of taxonomic affiliation of the genes on contigs which harbor a NCVOG which has potentially been subject to gene replacement or divergence after duplication. Each of the affected contigs is of viral origin based on gene content and can be attributed to the correct NCLDV lineage.

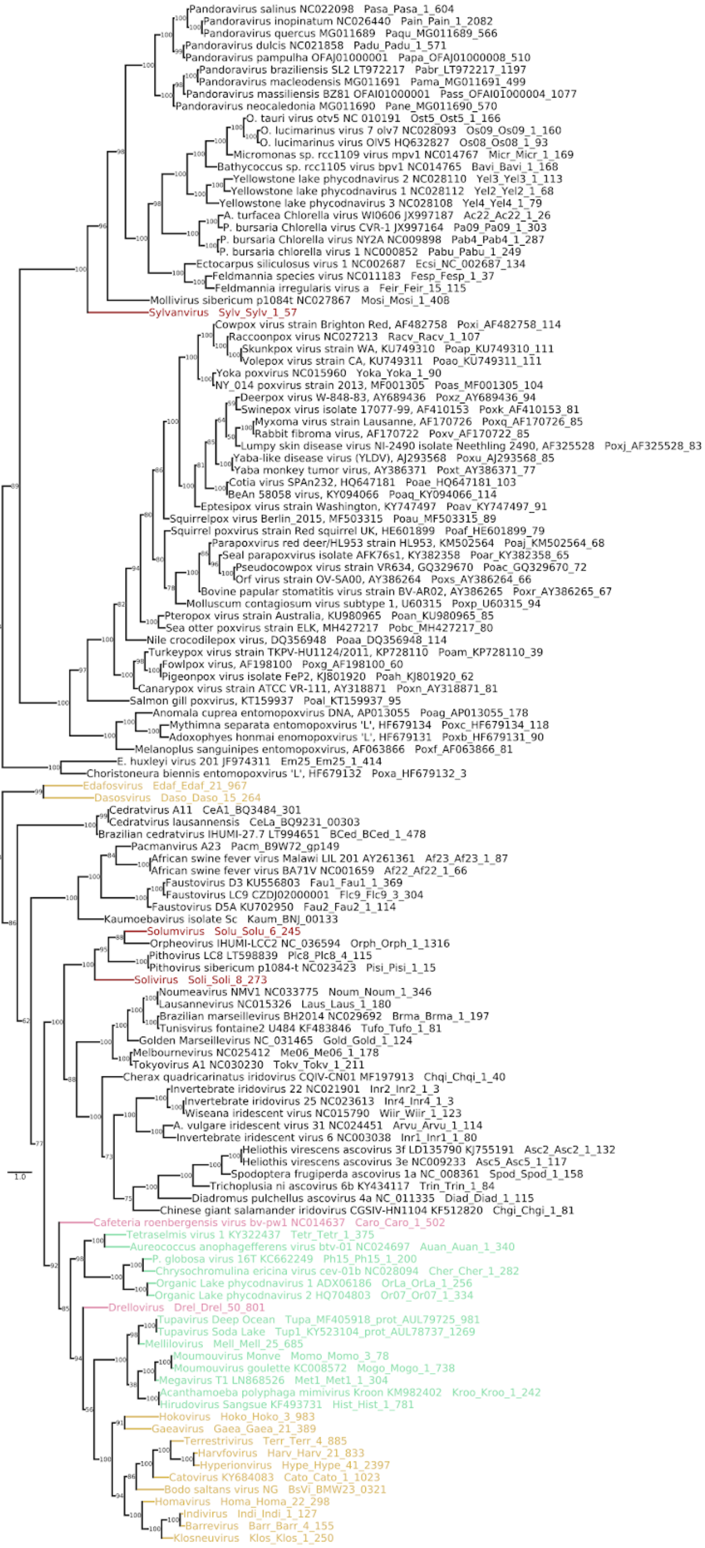

Supplementary Fig. 8a. Phylogenomic placement of novel viral lineages based on single protein phylogeny of D5-like helicase-primase (NCVOG0023). Maximum-likelihood tree was generated with IQ-tree using the best-fit model LG+F+R7 and 1000 ultrarapid bootstrap replications. The scale bar represents substitutions per site.

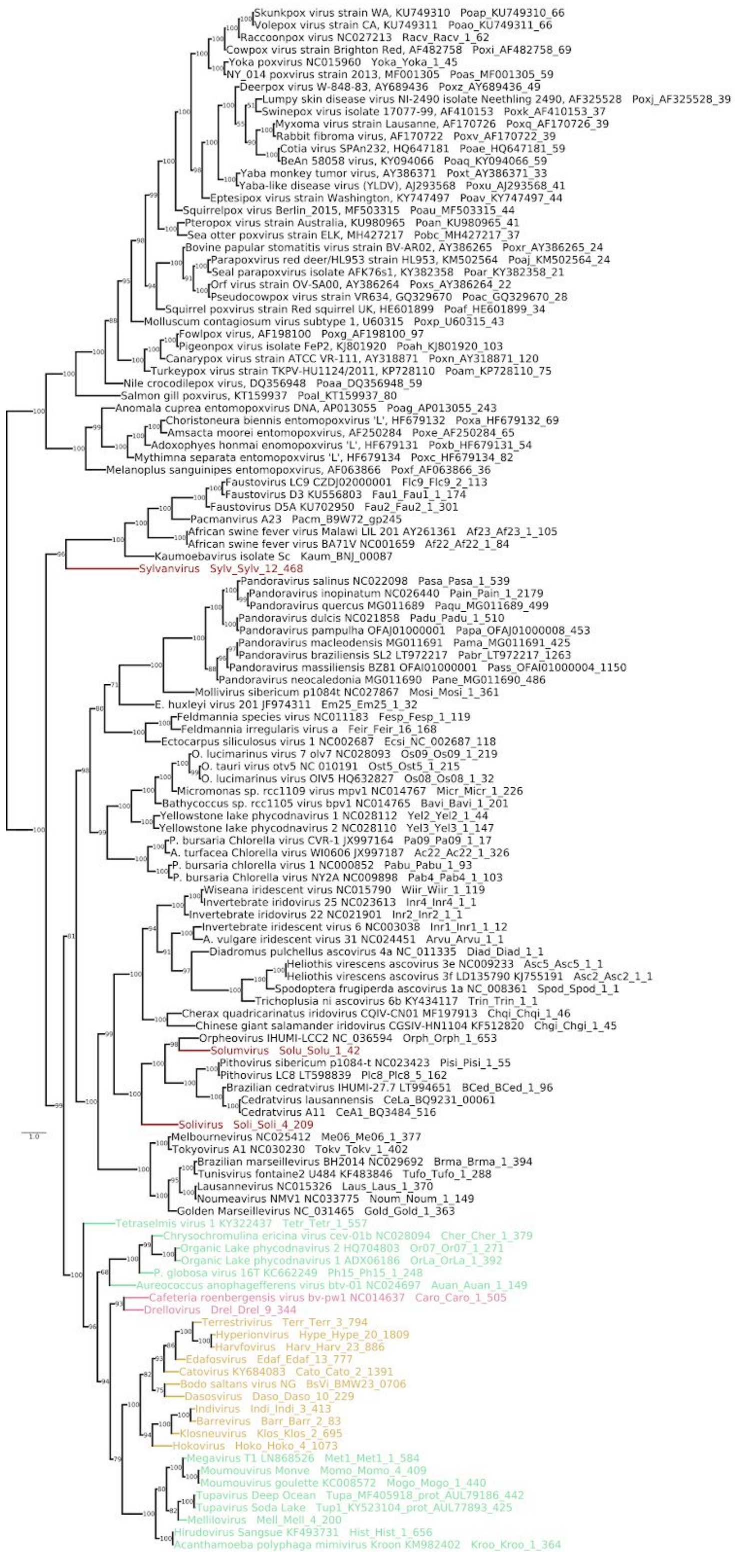

Supplementary Fig.8b. Phylogenomic placement of novel viral lineages based on single protein phylogeny of DNA polymerase elongation subunit family B (NCVOG0038). Maximum-likelihood tree was generated with IQ-tree using the best-fit model LG+F+R7 and 1000 ultrarapid bootstrap replications. The scale bar represents substitutions per site.

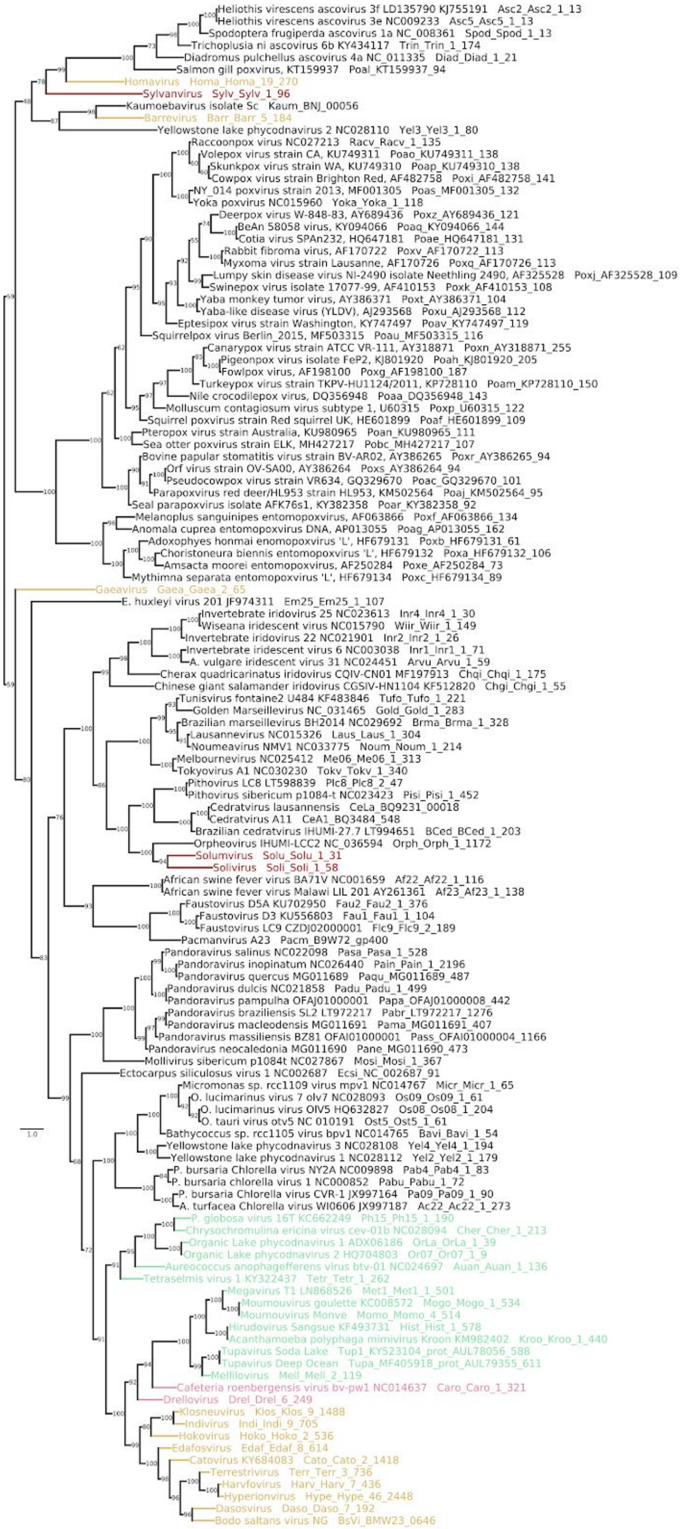

Supplementary Fig. 8c. Phylogenomic placement of novel viral lineages based on single protein phylogeny of DNA or RNA helicases of superfamily II (NCVOG0076). Maximum-likelihood tree was generated with IQ-tree using the best-fit model LG+F+R8 and 1000 ultrarapid bootstrap replications. The scale bar represents substitutions per site.

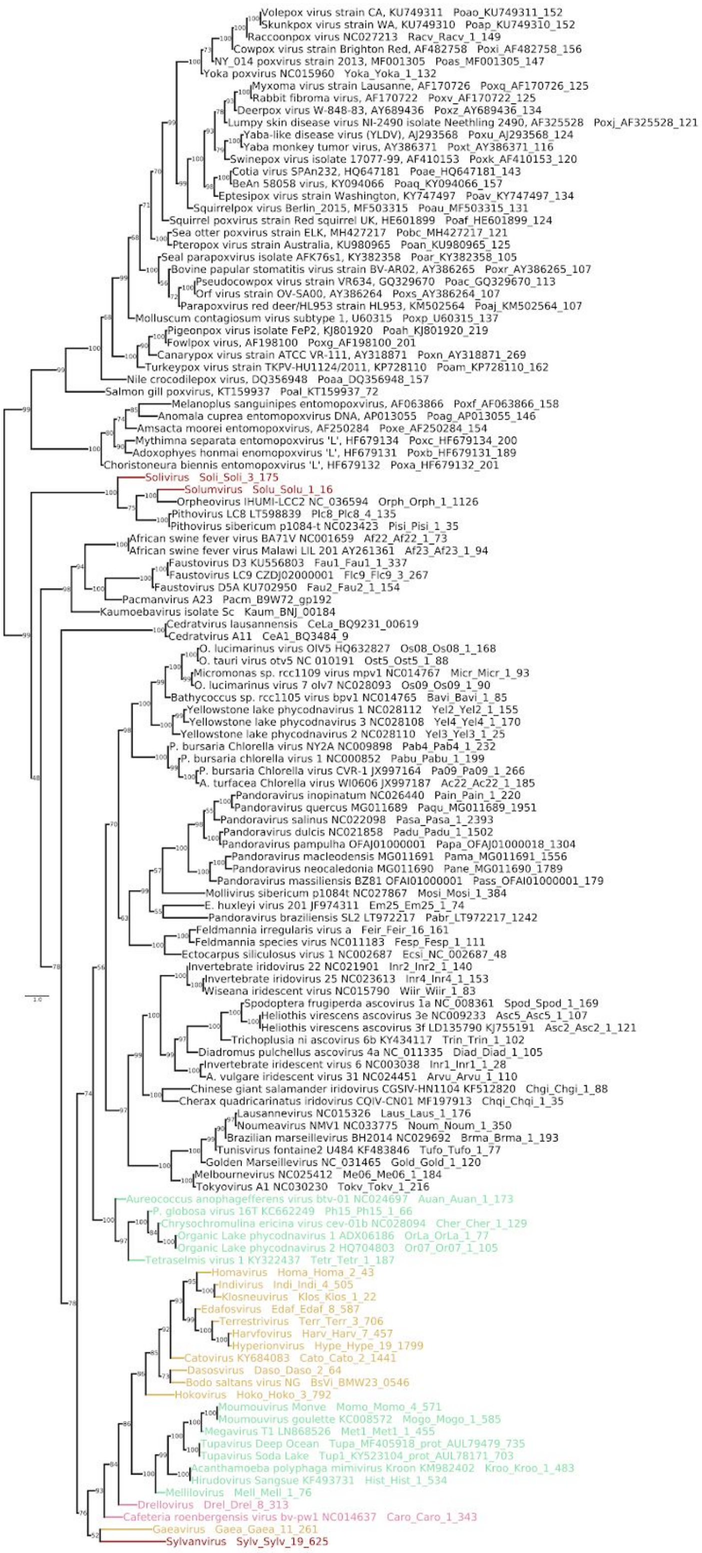

Supplementary Fig. 8d. Phylogenomic placement of novel viral lineages based on single protein phylogeny of packaging ATPase (NCVOG0249). Maximum-likelihood tree was generated with IQ-tree using the best-fit model LG+F+R5 and 1000 ultrarapid bootstrap replications. The scale bar represents substitutions per site.

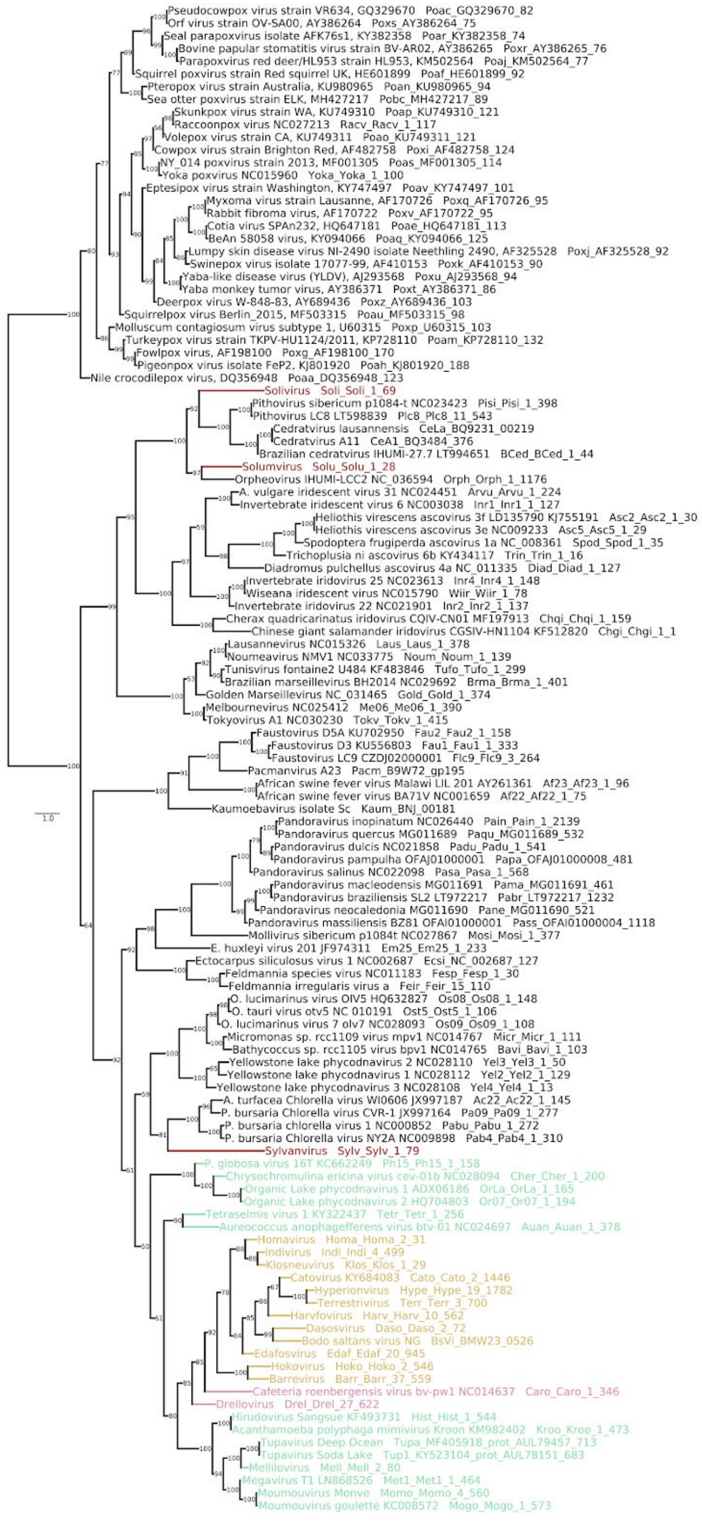

Supplementary Fig. 8e. Phylogenomic placement of novel viral lineages based on single protein phylogeny of Poxvirus Late Transcription Factor VLTF3-like (NCVOG0262). Maximum-likelihood tree was generated with IQ-tree using the best-fit model LG+F+R6 and 1000 ultrarapid bootstrap replications. The scale bar represents substitutions per site.

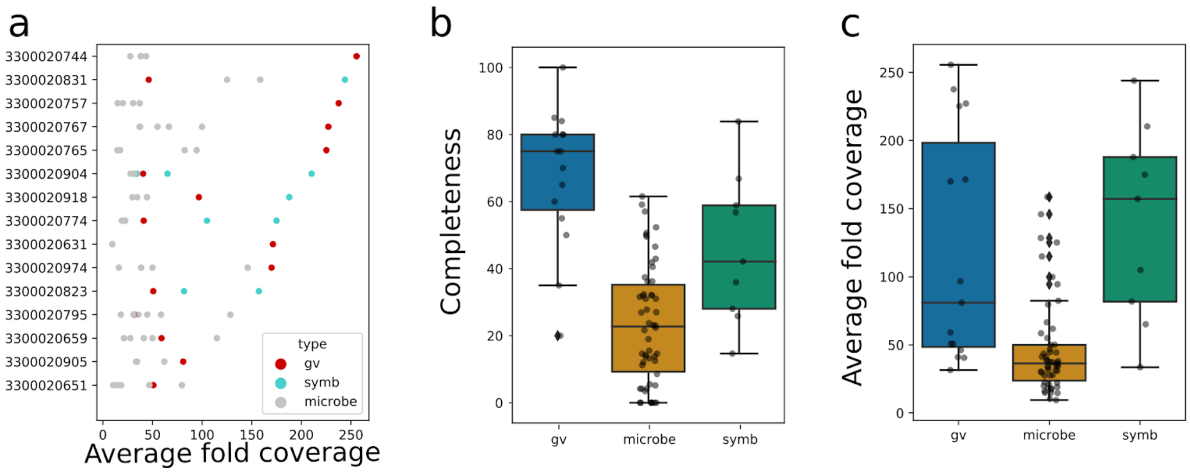

Supplementary Fig. 9. Average fold read coverage and completeness of MAGs derived from the mini-metagenomes in which the novel soil giant viruses were discovered. a Average fold mini-metagenome read coverage of MAGs in mini-metagenomes which contained giant virus MAGs. In most data sets the highest average read coverage was found in the giant virus MAGs and/or putative bacterial endosymbionts (MAGs affiliated with the microbial phylum *Dependentiae* or the bacterial order *Legionellales* or *Rickettsiales*), suggesting the presence of multiple identical viral particles or cells as template for multiple displacement amplification **b** Completeness estimate for giant virus MAGs (gv) based on total number of different conserved NCVOGs compared to related NCLDV genomes (Supplementary Fig. 1). Completeness of free-living microbes (microbe) and putative microbial symbionts (symb) provided by CheckM [7], which uses universally conserved genes to estimate completeness. Genomes of giant viruses and microbial endosymbionts which had the highest average MAG coverage tended to be the more complete than genomes of other microbes. **c** Summary of average fold read coverage of all MAGs contained in the mini-metagenomes in which the giant viruses were discovered. Center lines of boxplots represent the median, bounds of boxes the lower and upper quartile, whiskers extend to points that lie within 1.5 interquartile range of the lower and upper quartile.

Supplementary References

1. Yutin, N., Wolf, Y. I., Raoult, D. & Koonin, E. V. Eukaryotic large nucleo-cytoplasmic DNA viruses: clusters of orthologous genes and reconstruction of viral genome evolution. *Virol. J.* 6, 223 (2009).
2. Simmonds, P. *et al.* Consensus statement: Virus taxonomy in the age of metagenomics. *Nat. Rev. Microbiol.* 15, 161–168 (2017).
3. Kalvari, I. *et al.* Rfam 13.0: shifting to a genome-centric resource for non-coding RNA families. *Nucleic Acids Res.* 46, D335–D342 (2018).
4. Nawrocki, E. P. & Eddy, S. R. Infernal 1.1: 100-fold faster RNA homology searches. *Bioinformatics* 29, 2933–2935 (2013).
5. Quast, C. *et al.* The SILVA ribosomal RNA gene database project: improved data processing and web-based tools. *Nucleic Acids Res.* 41, D590–6 (2013).
6. Altschul, S. F. *et al.* Gapped BLAST and PSI-BLAST: a new generation of protein database search programs. *Nucleic Acids Res.* 25, 3389–3402 (1997).
7. Parks, D. H., Imelfort, M., Skennerton, C. T., Hugenholtz, P. & Tyson, G. W. CheckM: assessing the quality of microbial genomes recovered from isolates, single cells, and metagenomes. *Genome Res.* 25, 1043–1055 (2015).
